# Genome-wide identification and functional analysis of the WRKY transcription factor family in *Reynoutria japonica* reveals its role in balancing growth and abiotic stress tolerance

**DOI:** 10.1186/s12870-026-08480-3

**Published:** 2026-03-03

**Authors:** Fan-Hong Wang, Jia-Ying Wang, Ning-Ning Yang, Lan Luo, Qian Wu, Guo-Ying Wang, Wen-Rui Deng, Xiao-Wei Wang, Hong Wang

**Affiliations:** 1 College of Life Sciences, Northwest Normal University, Lanzhou, 730070 China; 2 College of Life Sciences, Institute of Life Sciences and Green Development, Hebei University, Baoding, 071002 China; 3 College of Life Sciences, University of Chinese Academy of Sciences, Beijing, 100049 China; 4 Tianjin Institute of Industrial Biotechnology, Chinese Academy of Sciences, Tianjin Airport Economic Area, No.32 West 7th Avenue, Tianjin, 300308 China

**Keywords:** Reynoutria japonica, WRKY gene family, Stem growth, Abiotic stress, RjWRKY11, Cadmium

## Abstract

*Reynoutria japonica*, known for its exceptional growth capacity and tolerance to multiple abiotic stresses, represents an ideal model for studying the trade-off between plant growth and stress adaptation. However, the molecular mechanisms underlying the balance between plant growth and stress resistance in *R. japonica* remain unclear. Increasing evidence indicates that WRKY proteins play a direct role in regulating the equilibrium between plant growth and resistance. In this study, we identified and conducted bioinformatics analysis about WRKY gene family from two haplotype chromosomes (HapA 89, HapE 82) in *R. japonica*. These 171 RjWRKY members distributed in three groups: group I (34), group II (115), and group III (22). RjWRKY members within the same group or subgroup typically exhibited similar conserved motifs and gene structures. Promoter sequences in the WRKY gene family contained a series of *cis*-acting elements related to plant growth and development, stress response, and hormone response. Based on transcriptome data, we screened 12 candidate *RjWRKY* genes that are associated with stem elongation and are simultaneously responsive to methyl jasmonate (MeJA, a stress-response ‘messenger’) and ultraviolet (UV) radiation. Further, we confirmed the relative expression levels of 12 *RjWRKY* genes during different stem-growth stages as well as under auxin (IAA) or auxin antagonist (PEO-IAA), salt, cadmium (Cd) and manganese(Mn) treatments by qRT-PCR. The 12 *RjWRKY* genes, responsive to both auxin signals and abiotic stress, may thus represent key regulators mediating the trade-off between plant growth and stress in this invasive plant species. In addition, overexpression of *HuZ00251595.1* (a homology of AtWRKY11, designated as RjWRKY11) in *A. thaliana* significantly enhanced resistance to Cd. These results establish a solid foundation for further exploration into the functions of the RjWRKYs in the balance of growth and adaptation to various abiotic stresses in *R. japonica*.

**Supplementary Information:**

The online version contains supplementary material available at 10.1186/s12870-026-08480-3.

## Introduction

*Reynoutria japonica* Houtt. (Polygonaceous) is a perennial Chinese herbal medicine. It is also a notorious invasive species, recognized for its vigorous growth capacity and strong stress tolerance [1, 2]. Plants’ growth and development are often influenced by various environmental factors, such as drought, metal stress, and salt stress. Under stress conditions, plants can balance the growth and abiotic stress by activating or repressing a range of genes [3–7]. However, research on the mechanisms balancing growth and stress tolerance in *R. japonica* has been hindered by its genomic complexity and high ploidy level (it is an octoploid) [8, 9].

The WRKY family represents one of the largest groups of TFs in plants and plays crucial roles in growth, development, metabolism, and stress responses, as well as in maintaining the equilibrium between growth and stress resistance [10, 11]. WRKY TFs contain typical WRKY domains (WRKYGQK) and a 60-amino acid zinc finger structure in the N-terminus and/or C-terminus [12, 13]. According to number of WRKY domains and the characteristic zinc finger motifs, the WRKY family can be divided into groups I, II and III [12]. Group I contains two WRKY domains and a C_2_H_2_ zinc finger motif; group II contains a WRKY domain and a C_2_H_2_ zinc finger motif and is further divided into five subgroups: IIa, IIb, IIc, IId, and IIe; group III consists of a WRKY domain and a C_2_HC zinc finger motif [13]. WRKY TFs specifically bind to the *cis*-elements W-box (TTGACC/T) on the promoter regions of target genes, thereby regulating their transcription [14, 15].

The number of WRKY family member in different species varies from 38 to 180 [10]. An increasing number of studies indicate that WRKY TFs are involved in plant growth and development [16–18]. Meanwhile, studies have demonstrated that WRKY TFs also play a crucial role in responding to specific abiotic stresses such as drought, metal stress, and salt stress [19–22]. Overall, *WRKY* genes are involved in plant growth and development as well as exhibiting effective responses or tolerances to various abiotic stresses in different plants (Table S1).

Therefore, identifying WRKY genes that coordinate both growth and stress tolerance in *R. japonica*, and elucidating their balancing mechanisms, is of significant interest. The high-quality and chromosome-scale genome of the *R. japonica* has enabled the identification and characterization of the WRKY gene family. Here, we systematically identify and characterized the WRKY gene family at the genome-wide level with haplotypes A and E (HapA and HapE) representing two subgenome. The phylogenetic relationship, conserved motif, chromosomal location, and gene structure of RjWRKYs were comprehensively analyzed. Furthermore, we selected 12 genes involved in stem elongation that were also implicated in the response to MeJA and UV stress. The expression profiles of these genes were confirmed by qRT-PCR under hormone (IAA and PEO-IAA), salt, cadmium (Cd) and manganese(Mn) treatments stress. We overexpressed *HuZ00251595.1* (the homolog of AtWRKY11, here named as RjWRKY11) in *A. thaliana* and investigated its Cd tolerance. This work provides a foundation for understanding the biological functions of *RjWRKY* genes and explores their potential roles in regulating the trade-off between plant growth and stress resistance.

## Materials and methods

### Plant materials

*R. japonica* seeds collected from the Beijing Botanical Garden were planted on Murashige and Skoog (MS) medium in light incubator. The cultrue conditions were set to: relative humidity 60%, temperature 22 ± 1 °C, 16/8 h light and dark cycle. After two months of growth, uniform seedlings were transferred to Hoagland nutrient solution for hydroponic cultivation and allowed to acclimate for three weeks prior to treatment. For salt stress, these seedling were treated with 200 mM NaCl for 0, 6, 12, and 24 h. For heavy stree, these seedling were subjected to 150 µM CdSO_4_ or MnSO_4_ solutions for 24 h and 7d, respecttively. Meanwhile these seedling were treated with 100 µM IAA or 10 µM PEO-IAA for 1 h and 3 h, respectively. The untreated seedlings were used as the control (0 h). Underground and aboveground parts were collected, respectively, and quickly frozen in liquid nitrogen, and stored at -80 °C. Transgenic *A. thaliana* (Columbia ecotype, Col-0) seeds were planted on 1/2 MS medium under the following conditions: relative humidity 50%, temperature 22 ± 1℃, 16/8 h light and dark cycle.

### Identification of the WRKY gene family in *R. japonica* genome

The *R. japonica* genome sequence file, gff file, protein sequence and coding sequence (CDS) data used in this paper are unpublished.

We obtain RjWRKY member using BlastP and Hidden Markov Model (HMM). Firstly, the 72 AtWRKY protein sequences in *A. thaliana* from the TAIR database (https://www.Arabidopsis.org) were aligned with the whole-genome data of *R. japonica* using BlastP to obtain candidate WRKY members. Secondly, HMM file for the WRKY TF domain (PF03106) was obtained from the Pfam protein family database (http://pfam.xfam.org/) and WRKY members were searched in the *R. japonica* genome based on the HMM method. The preliminary sets of both have been submitted to NCBI-CDD (https://www.ncbi.nlm.nih.gov/cdd/) and Pfam (http://pfam.xfam.org/) for domain verification. The final RjWRKYs were reserved after deleting sequences containing incomplete domains or not containing domains. The ExPASy (https://www.expasy.org/) was used to analyze the amino acid sequences of RjWRKYs for pI (isoelectric point), MW (molecular weight), amino acid count, and GRAVY (grand average of hydropathicity). The subcellular localization was predicted using Plant-mPLoc (http://www.csbio.sjTu.edu.cn/bioinf/plant-multi/). The transmembrane region was predicted using TMHMM-2.0 (https://services.healthtech.dtu.dk/services/TMHMM-2.0/).

### Phylogenetic analysis and classification of RjWRKY genes

To study the phylogeny and classification of the RjWRKY gene family, MEGA7 software [23] was used to construct a phylogenetic tree based on protein sequences of AtWRKY and RjWRKY using the neighbor-joining (NJ) method with 10,000 bootstrap repetitions. Subsequently, iTOL (https://itol.embl.de) was uesd to improve the phylogenetic tree. The multiple sequence alignment of RjWRKYs was performed using Clustalx software and was visualized using Genedoc software.

### Chromosome localization, gene structure and conserved motif analysis of RjWRKYs

The chromosome position information of RjWRKY gene family members were extracted from the *R. japonica* genome GFF file, and TBtools software [24] was used to visualize the chromosome positions. Gene structures were analyzed using TBtools software. Conserved motif prediction of RjWRKY sequences was performed using the MEME (http://meme-suite.org) website, with the number of motifs set to 10.

### Analysis of *cis*-acting elements in promoter region of *RjWRKY* gene

Upstream 2,000 bp sequences of the transcription start site of the *RjWRKY* genes were obtained from the *R. japonica* genome. Then, the *cis*-elements of promoter was analyzed using Plant CARE website (http://bioinformatics.psb.ugent). Finally, the *cis*-elements were visualized by TBtools.

### Analysis of expression patterns of *RjWRKY* genes

Transcriptome data in *R. japonica* root, stem, and leaf (Genbank ID: PRJNA623335) as well as under MeJA treatment (0, 8, and 16 h; Genbank ID: PRJNA626400) and UV treatment (0, 6, and 12 h; Genbank ID: PRJNA451292) were downloaded and the FPKM of RjWRKY family genes was extracted and used to draw heat maps using TBtools software. We utilized transcriptome data from stem tissues at the elongation stage and from plants subjected to MeJA and UV treatments to represent growth and abiotic stress-related conditions, respectively, in *R. japonica*. Based on these datasets, potential 12 *RjMYB* genes involved in regulating growth and stress responses were screened. Subsequently, the expression patterns of the candidate *RjMYB* genes were further validated under growth-related conditions (IAA and auxin PEO-IAA) and multiple abiotic stresses (Cd, Mn, and NaCl).

### qRT-PCR analysis of *RjWRKY* genes

The RNA was extracted using the Trizol method (Trizol reagent). The Premier 5 was used to design primer sequences for 12 candidate *RjWRKY* genes (Table S2). First, one-step cDNA synthesis was performed and then qRT-PCR reaction was performed using FastKing gDNA Dispelling RT SuperMix and FastReal qPCR PreMIX (SYBR Green) from TIANGEN (BIOTECH Beijing CO., LTD) according to the instructions. *RjGAPDH* (*HuZ00253337.1*) gene was used as the internal reference gene with forward primer: ACAGTTCACGCAATGACCGC and reverse primer: TGCTGCTGGGAATGATGTTG. The qRT-PCR cycling conditions are: 95 °C for 2 min, 95 °C for 5 s, 60 °C for 30 s, with a total of 40 cycles. The system utilizes the 2^−△△Ct^ method to normalize the expression levels of the candidate gene.

### Metal resistance analysis

The overexpression lines of RjWRKY11 in *A. thaliana* were used as described in the.

previous study [25]. The T3 generation seeds of transgenic lines (OE1, OE2, and OE3, randomly selected) and wild-type (WT) seeds were planted on 1/2 MS (50 mg/L Kan) medium to germinate and grow for 4 days, and then the seedlings were transferred to 1/2 MS (**Control**, 40 µM, 60 µM, and 80 µM CdSO_4_) medium to grow vertically for 7 days. The growth status of three transgenic lines and WT was observed, and their root length and fresh weight were measured and weighted.

## Results

### Identification and analysis of the WRKY gene family in *R. japonica*

We used the HMM and BlastP method to finally identify 171 RjWRKY members (HapA: 89, HapE: 82) from the two haplotypes (A and E) of *R. japonica*. The amino acid lengths of 171 RjWRKY ranged from 92 amino acids (HuZ00224110.1) to 824 amino acids (HuZ0098955.1), with an average protein sequence length of 366 amino acids. The relative molecular weights ranged from 9.77 kD (HuZ00224110.1) to 90.86 kD (HuZ0098955.1), and the isoelectric points(pI) ranged from 5.02 (HuZ0040851.1) to 9.99 (HuZ0039975.1). The hydrophilicity analysis results revealed that all RjWRKY proteins exhibited negative GRAVY values with an average hydrophilicity of -0.72, indicating they are hydrophilic proteins. The instability coefficients ranged from 34.5 (HuZ00224110.1) to 77.62 (HuZ0020860.1), with 12 members having instability coefficients (less than 40). The aliphatic index ranged from 40.33 (HuZ00130781.1) to 99.67 (HuZ00224110.1). Subcellular localization prediction results indicated that all 171 RjWRKY proteins were localized in the cell nucleus, and this positional distribution may be adapted to the functions of these proteins in the cell (Table S3).

### Phylogenetic analysis of RjWRKYs

**Fig. 1:**
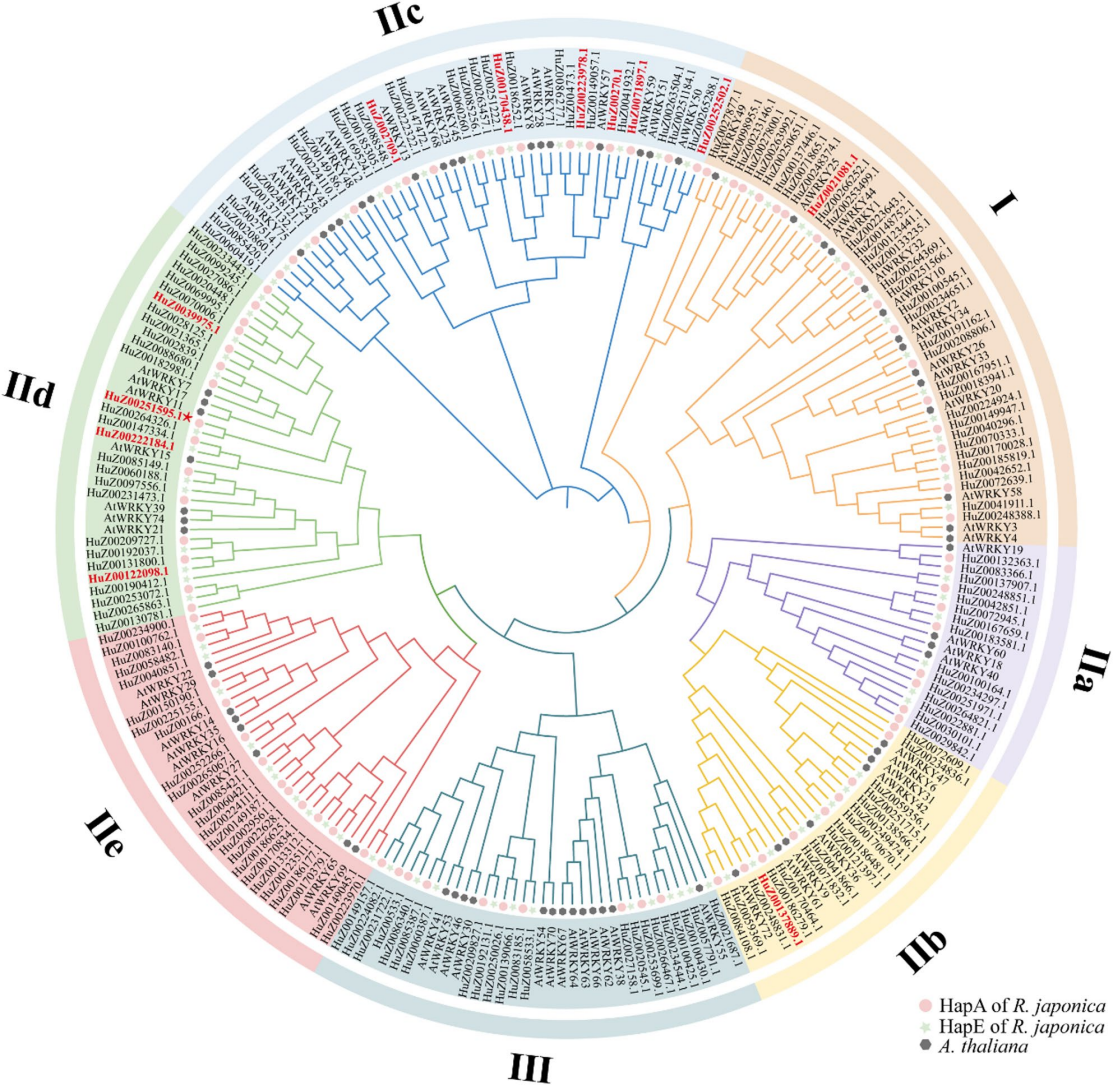
Phylogenetic tree of WRKY proteins from *R. japonica* and *A. thaliana. * Red dots, green pentagons and black polygons represent WRKY members of R. japonica HapA, HapE and A. thaliana, respectively. Roman numerals I, II, and III represent three groups, with a-e representing subgroup of group Ⅱ. The red IDs represent 12 candidate RjWRKY genes and the asterisk (*) represents RjWRKY11 (HuZ00251595.1)

Based on the evolutionary tree of 245 WRKYs from *A. thaliana* and *R. japonica* (HapA and HapE), three groups were divided: group I (34; 18 for HapA, 16 for HapE), group II (115; 61 for HapA, 54 for HapE), and group III (22; 10 for HapA, 12 for HapE) (Fig. 1). Group II can be further subdivided into five subgroups (IIa, IIb, IIc, IId, and IIe) containing 15, 17, 31, 28, and 24 members, respectively. This classification corresponds to the grouping of WRKY families from other plants [26]. These results may help predict the functions of unknown RjWRKYs based on the roles confirmed in AtWRKYs or their subgroups.

**Fig. 2:**
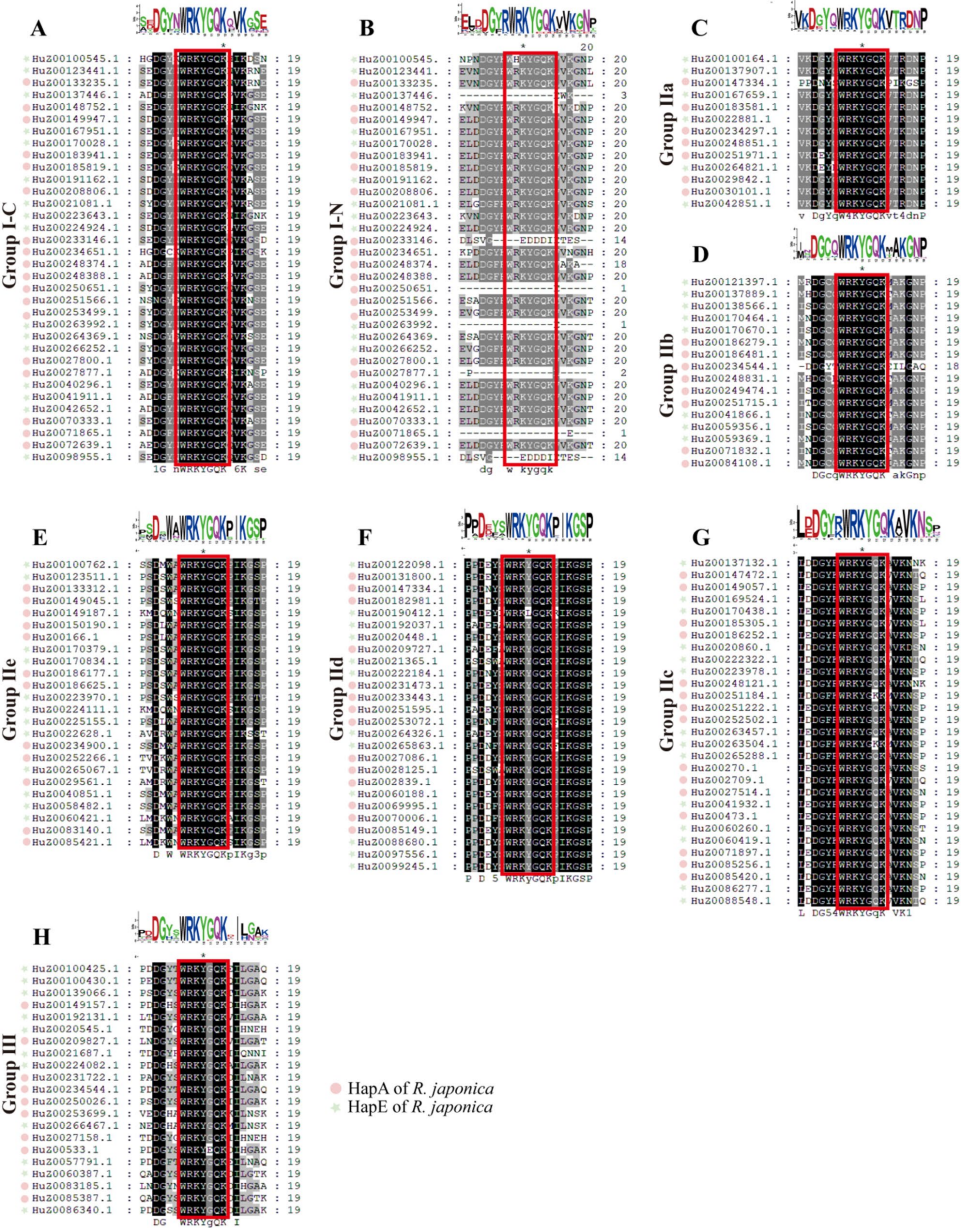
Multiple sequence alignment of domains of RjWRKY family members. **A**-**H** represent the result of multiple sequence alignment of three group (I, II, and III) and five subgroup members of WRKY family in R. japonica, respectively. Red dots and green pentagons represent WRKY members of R. japonica HapA and Hap, respectively. WRKY domains were displayed in the red box. I-N and I-C represent the N-terminal and C-terminal WRKY domains of the family, respectively

79.4% (164/171) RjWRKY members contain the highly conserved WRKYGQK motif and the other seven genes (20.6%, group I) have an N-terminal deletion (Fig. 2). In addition, six RjWRKY members have an amino acid residue substitution in WRKYGQK motif: WRKYGKK, WKKYGQK, WHKYGQK, WRKYEQK, and WRKLGQK (Fig. 2).

### Chromosome localization, gene structure and conserved motif analysis of RjWRKYs

**Fig. 3:**
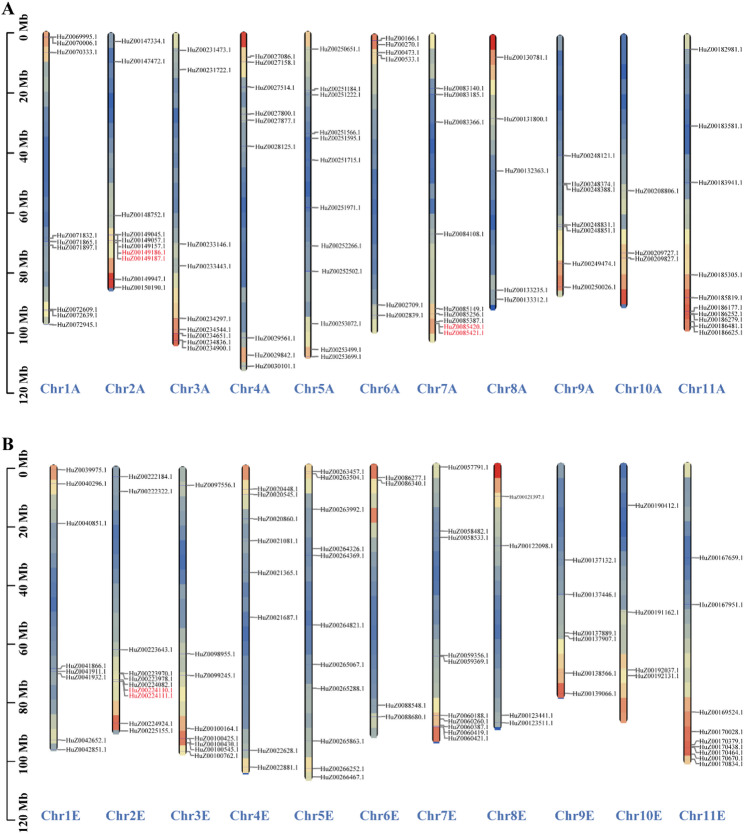
The location of *RjWRKY* genes in HapA and HapE chromosomes. **A** Distribution of RjWRKY genes on HapA chromosomes. **B** Distribution of RjWRKY genes on HapE chromosomes. The red gene IDs indicate the tandem repeat pairs of RjWRKY genes on the chromosomes

Chromosomal localization analysis reveals that the 171 *RjWRKY* genes are distributed across the 22 chromosomes (Chr.) of the HapA and HapE, with genes from the same subgroup randomly distributed on the chromosomes (Fig. 3). Chr. 5 carries the highest number of *RjWRKY* genes (23; 12 for HapA, 11 for HapE), while Chr. 10 contains the fewest number of *RjWRKY* genes (7; 3 for HapA, 4 for HapE). Chr. 1 and Chr. 6 have the same number of genes in HapA and HapE. Chr. 7 and Chr. 10 have more genes in HapE than in HapA. In contrast, HapA contains more *RjWRKY* members than HapE in 7 chromosomes (Chr. 2, Chr. 3, Chr. 4, Chr.5, Chr.8, Chr.9, and Chr. 11) (Table S4). If a chromosomal region contains two or more genes within the 200 kb range, that chromosomal region is defined as having experienced a tandem replication event [27]. We identified three tandem repeat events involving six *RjWRKY* genes on the HapA and HapE chromosomes. Namely, two pairs of genes tandem repeats in HapA (*HuZ00149186.1/HuZ00149187.1* and *HuZ0085420.1*/*HuZ0085421.1*) and one tandem duplicated gene pair in HapE (*HuZ00224111.1*/*HuZ00224110.1*) (Fig. 3).

**Fig. 4:**
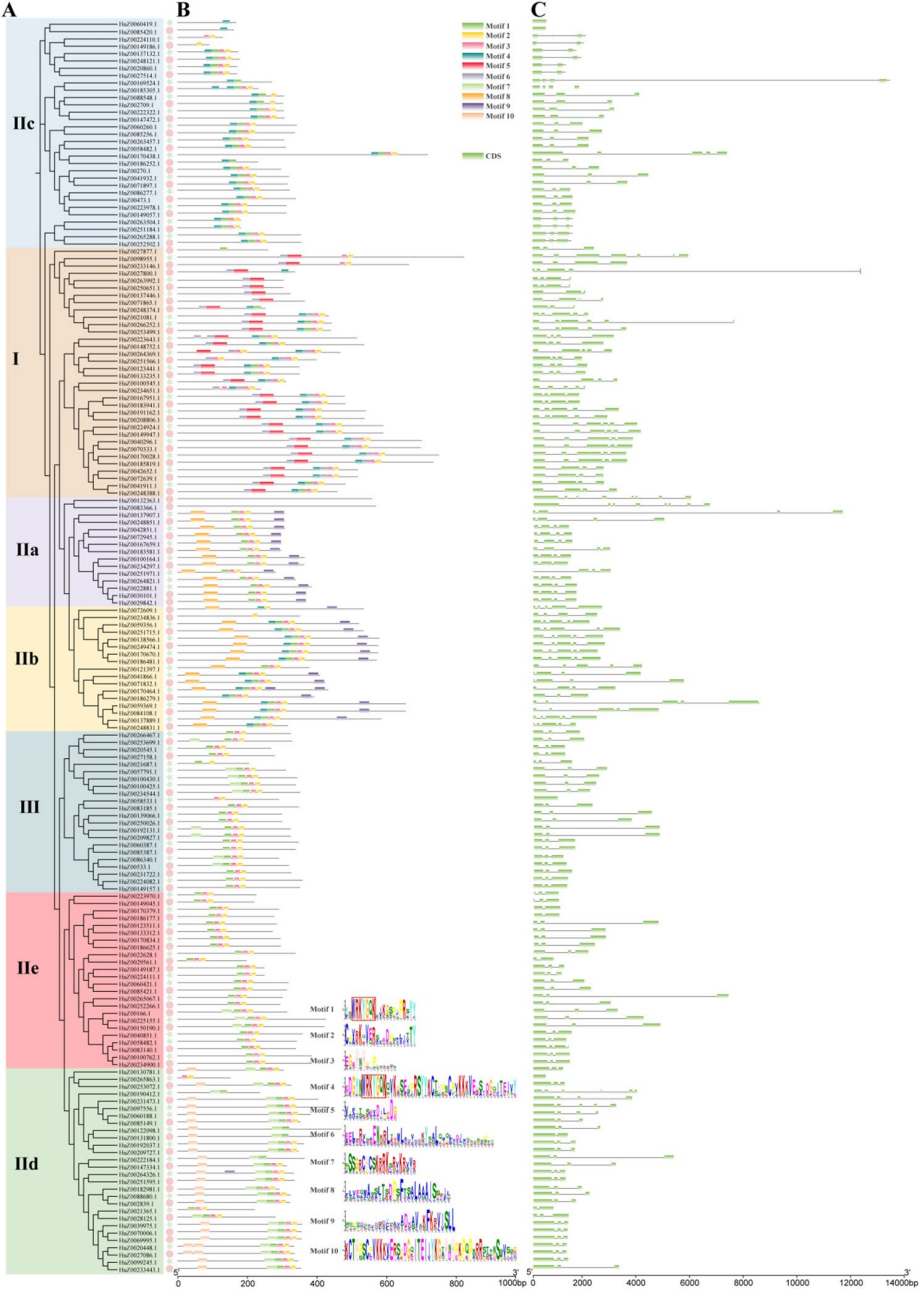
Phylogenetic relationships, motifs, and gene structures of RjWRKY family members. **A** A phylogenetic tree of 171 RjWRKY proteins. **B** Conserved motifs of RjWRKYs. **C** Exon-intron structures of RjWRKY genes

The conserved motifs results show that 169 RjWRKY members contain 1–8 motifs, and RjWRKYs within the same group or subgroup have similar motif compositions (Fig. 4A-B). Motif 1 and motif 4 compose WRKY domains. In detail, motif 4 only exists in group I, subgroup IIb, and subgroup IIc, while motif 1 is distributed in all groups. Motif 5 and motif 6 are exclusively present in group I, motif 8 and motif 9 are exclusively present in subgroup IIa and subgroup IIb, motif 10 is found only in subgroup IId, indicating that these three motifs may possess specific roles (Fig. 4A-B). These specific motifs are distributed in the same subgroup of the evolutionary tree and further support the division of three groups and five subgroups of RjWRKY.

All 171 *RjWRKY* genes have both exons and introns, with the number of introns ranging from two to eight (Fig. 4C). The genes in group I contain two to six introns, with 44.11% of the genes having three introns. Among the 83 *RjWRKY* genes in subgroups IIc, IId, and IIe, 75.9% (62/83) *RjWRKY* genes contain two introns. More introns were observed in subgroups IIa and IIb, with 11 *RjWRKY* genes containing three and four introns, respectively. In group III, all genes have two introns except for *HuZ0058533.1* (one intron). The exon-intron structure of the 171 *RjWRKY* genes correspond to the cluster of the three groups and five subgroups in evolutionary tree.

### Analysis of *cis*-acting elements in promoter region of *RjWRKY* genes

**Fig. 5:**
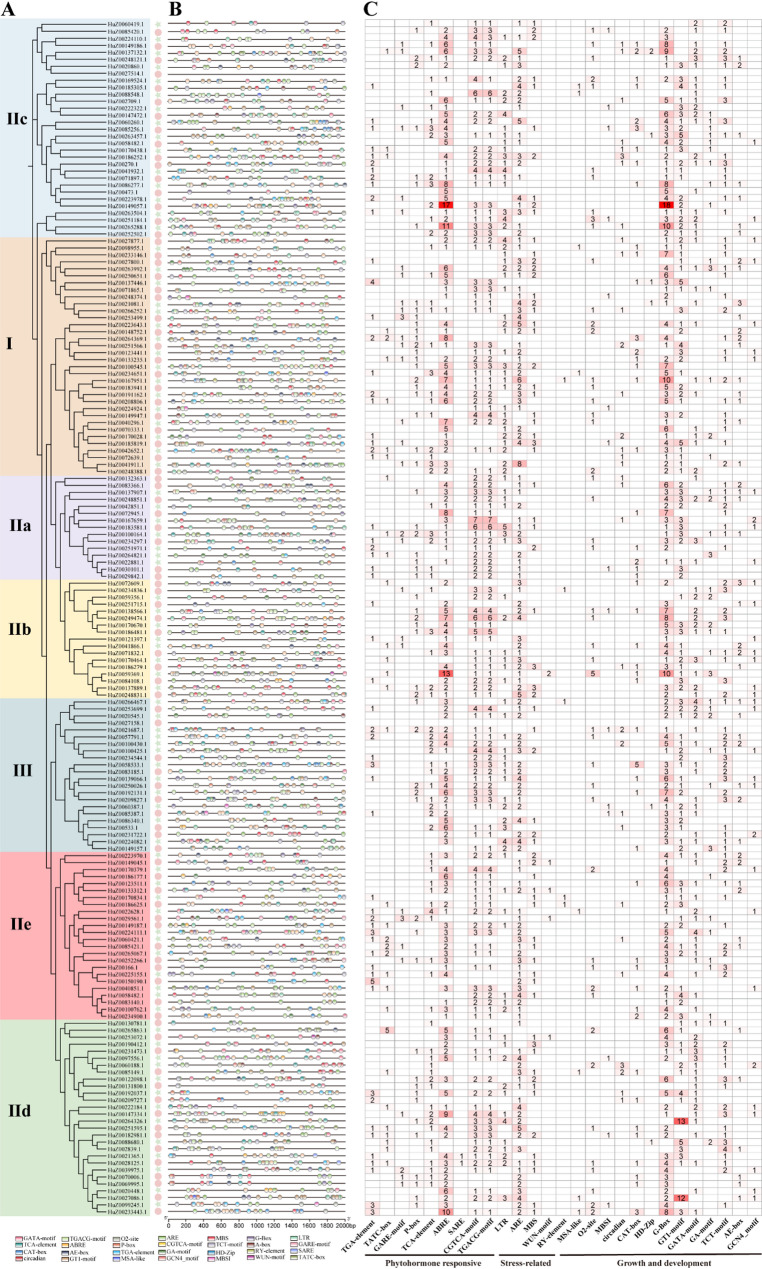
Analysis of cis-elements in the promoter regions of *RjWRKY* genes. **A** A phylogenetic tree of 171 RjWRKY proteins. **B** Location of the distribution of cis-acting elements of RjWRKY genes. **C** The number of different cis-acting elements of RjWRKY genes. The different colored blocks represent the different types of cis-elements and their locations in each RjWRKY gene. Different colors and numbers in the grid indicate the numbers of different promoter elements in the RjWRKY genes

Among the promoter regions of 171 *RjWRKY* genes, various *cis*-acting elements were discovered in 169 *RjWRKY* genes. The three classes of *cis*-acting elements associated with plant hormone response stress response, and growth and development were summarized and sporadically distributed in the promoter regions of *RjWRKY* genes (Fig. 5). The first category comprised of plant hormone-responsive *cis*-acting elements, including Abscisic Acid Response Elements (ABREs), Auxin-responsive Element (TGA-element) Gibberellin-Responsive Elements (GARE-motif, P-box, and TATC-box), Jasmonic Acid Response Elements (CGTCA-motif and TGACG-motif), and Salicylic Acid Response Elements (TCA-element and SARE). These results indicate that *RjWRKY* genes are regulated by multiple hormones. The second category is stress-related *cis*-acting elements in the promoter regions of *RjWRKY* genes: Low-Temperature Responsive Element (LTR), Anaerobic Induction-Responsive Element (ARE), Drought-Inducible Element (MBS), Salt Stress-Inducible Element (ABRE), and Wounding Response Element (WUN-motif). The third category includes *cis*-acting elements related to plant growth and development, including endosperm expression (GCN4_motif), meristem expression (CAT-box), MADS-Box binding site I (MBSI), light-related elements (G-box). The promoter regions of 91.0% (81/89) and 95.12% (78/82) *RjWRKY* genes from HapA and HapE, respectively, contained *cis*-acting elements related to stress response and the promoter regions of 96.6% (86/89) and 100% (82/82) *RjWRKY* genes contained *cis*-acting elements related to plant growth and development, respectively. In summary, the promoters of the vast majority of RjWRKY genes are simultaneously enriched with cis-elements related to growth, development, hormones, and stress responses, providing genomic-level support for the hypothesis that RjWRKY transcription factors may play important roles in coordinating plant growth/development and stress adaptation.

### Analysis of expression patterns of *RjWRKY* genes during stem elongation

**Fig. 6:**
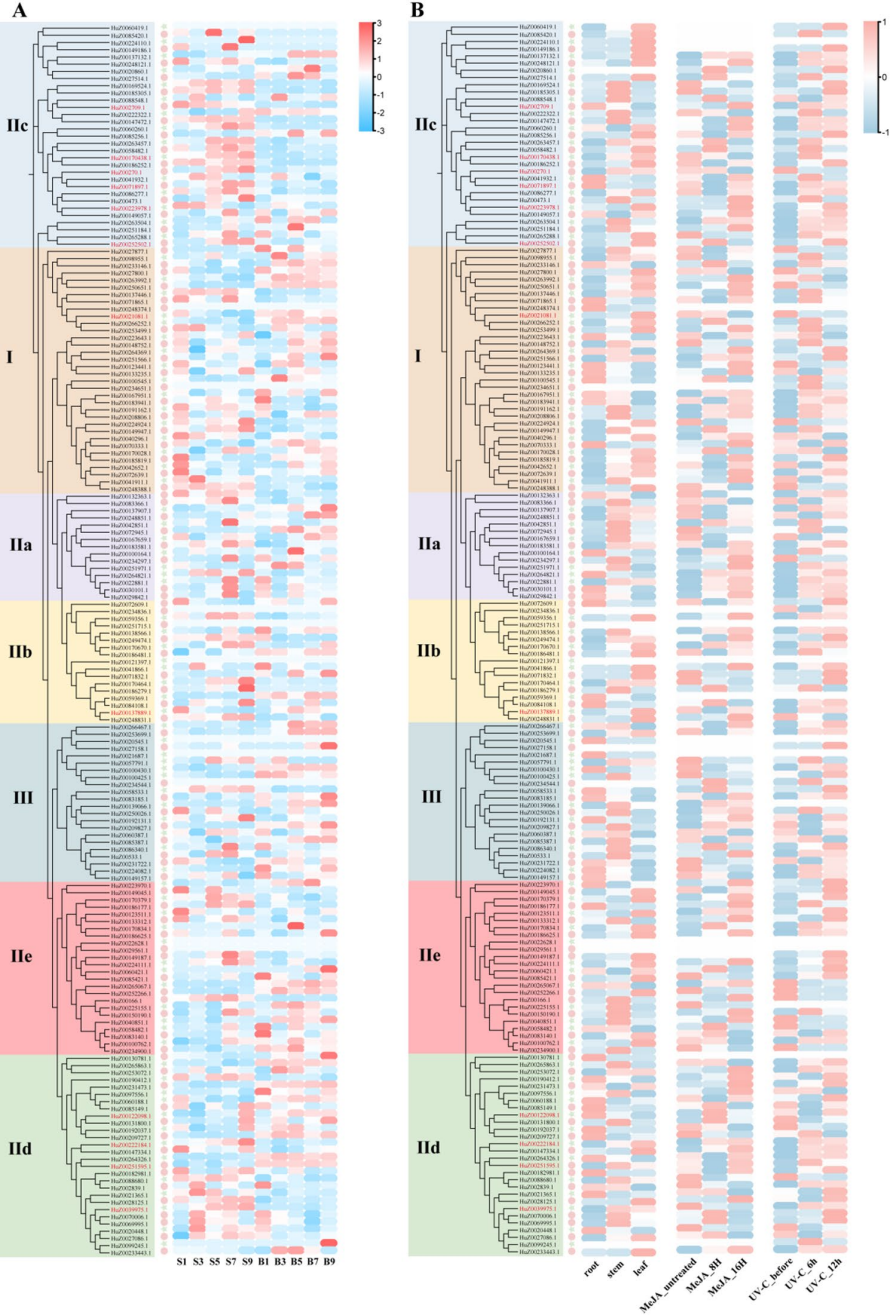
Expression profiles of all *RjWRKY* genes at different growth stages and abiotic stress treatments. **A** The heatmap of expression for 171 RjWRKY genes in five stem tips and internodes with the different stem length representing different growth stage. **B** The heatmap of expression for 171 RjWRKY genes in different tissues, under MeJA treatment, and UV treatment. S, stem internode; B, stem tips. The red IDs represent 12 candidate RjWRKY genes

Using RNA-seq data [28] from stem internodes (S) and stem tips (B) during different growth stages in *R. japonica*, the expression profiles of 171 *RjWRKY* genes were analyzed (Fig. 6A). The expression levels of 159 and 151 *RjWRKY* genes varied during stem elongation in the internodes and tips, respectively. The expression levels of 93.25% (83/89) and 92.68% (76/82) *RjWRKY* genes from HapA and HapE, respectively changed with the elongation of stem internodes. Among those, the expression levels of 12 *RjWRKY* genes positively or negatively respond stem elongation growth. In detail, the expression level of seven *RjWRKY* genes (*HuZ0039975.1*, *HuZ0071897.1*, *HuZ00222184.1*, *HuZ00122098.1*, *HuZ00252502.1*, *HuZ00270.1*, and *HuZ00170438.1*) increased with stem-internodes elongation, indicating that these RjWRKYs were able to promote stem-length elongation growth. In contrast, the expression level of five *RjWRKY* genes (*HuZ00137889.1*, *HuZ0021081.1*, *HuZ00223978.1*, *HuZ00251595.1*, and *HuZ002709.1*) decreased with stem-internodes elongation, suggesting that these RjWRKYs may have a negative effect on stem-length growth (Fig. 6B).

**Fig. 7:**
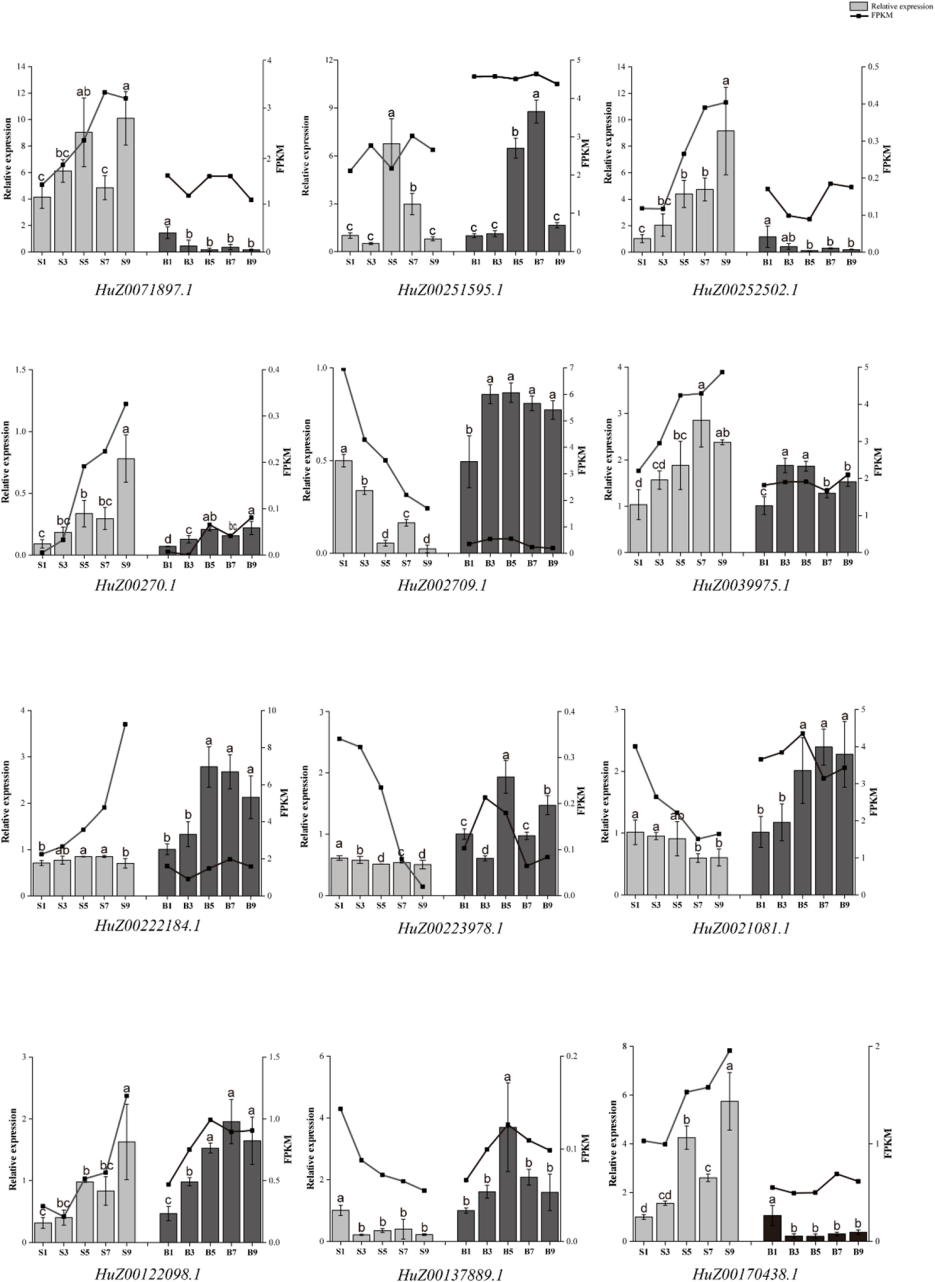
The relative expression level of 12 candidate *RjWRKY* genes at different growth stage. The error bars show the mean ± SE of three technical replicates. Black and gray histograms show qRT-PCR data from five stem internodes and stem tips, respectively, and the broken line graph indicates FPKM data. S, stem internode; B, stem tips. Letters (a, b, c, and d) indicate significant differences at five growth stages (Tukey-Kramer test, p < 0.05)

To validate the stem elongation-associated expression patterns of these 12 *RjWRKY* genes, we performed qRT-PCR on both stem internodes (S) and stem tips (B) across five developmental stages. The expression trends of 10 genes (*HuZ0039975.1*, *HuZ00122098.1*, *HuZ00252502.1*, *HuZ00270.1*, *HuZ00170438.1*, *HuZ00137889.1*, *HuZ0021081.1*, *HuZ00223978.1*, *HuZ002709.1* and *HuZ00222184.1*) were generally consistent with that in the transcriptome data. Specifically, six genes exhibited gradually increasing expression levels with continued internode elongation, whereas the other four showed a decreasing trend (Fig. 7). It is worth noting that the expression trend of two genes (*HuZ00251595.1* and *HuZ0071897.1*) from qRT-PCR was not consistent with results of transcriptome data. Their expression levels increased at stages S3, S5, and S7 compared to S1 but decreased from S7 to S9 (Fig. 7).

### Expression profiling of *RjWRKY* genes under abiotic stresses and growth condition

Jasmonic acid (JA) and its derivatives MeJA, act as key signaling molecules in plant stress responses and also influence growth processes. WRKY proteins (such as OsWRKY53) can simultaneously regulate stress responses (such as activating the JA/SA pathways) and growth development (such as influencing GA or IAA synthesis). To further observe the expression pattern of all *RjWRKY* genes under different abiotic stresses. The expression profile of *RjWRKY* genes under two abiotic stresses (MeJA and UV) was analyzed [29]. The results showed that 153 and 159 *RjWRKY* genes responded to MeJA and UV treatment, respectively. Of these, 88.76% (79/89) and 90.24% (74/82) *RjWRKY* genes in HapA and HapE, respectively, were responsive to MeJA treatment and 95.5% (85/89) and 90.24% (74/82) *RjWRKY* genes in HapA and HapE, respectively were responsive to UV treatment. Among the 12 stem elongation‑associated genes, six (*HuZ002709.1*, *HuZ00223978.1*, *HuZ0021081.1*, *HuZ00137889.1*, *HuZ00222184.1*, and *HuZ00122098.1*) were upregulated under both MeJA and UV treatments compared with the control (CK), whereas two (*HuZ00270.1* and *HuZ0021081.1*) were downregulated. The remaining four genes (*HuZ00170438.1*, *HuZ0071897.1*, *HuZ0039975.1*, and *HuZ00251595.1*) showed variable responses, being either induced or repressed under MeJA or UV stress (Fig. 6C).

**Fig. 8:**
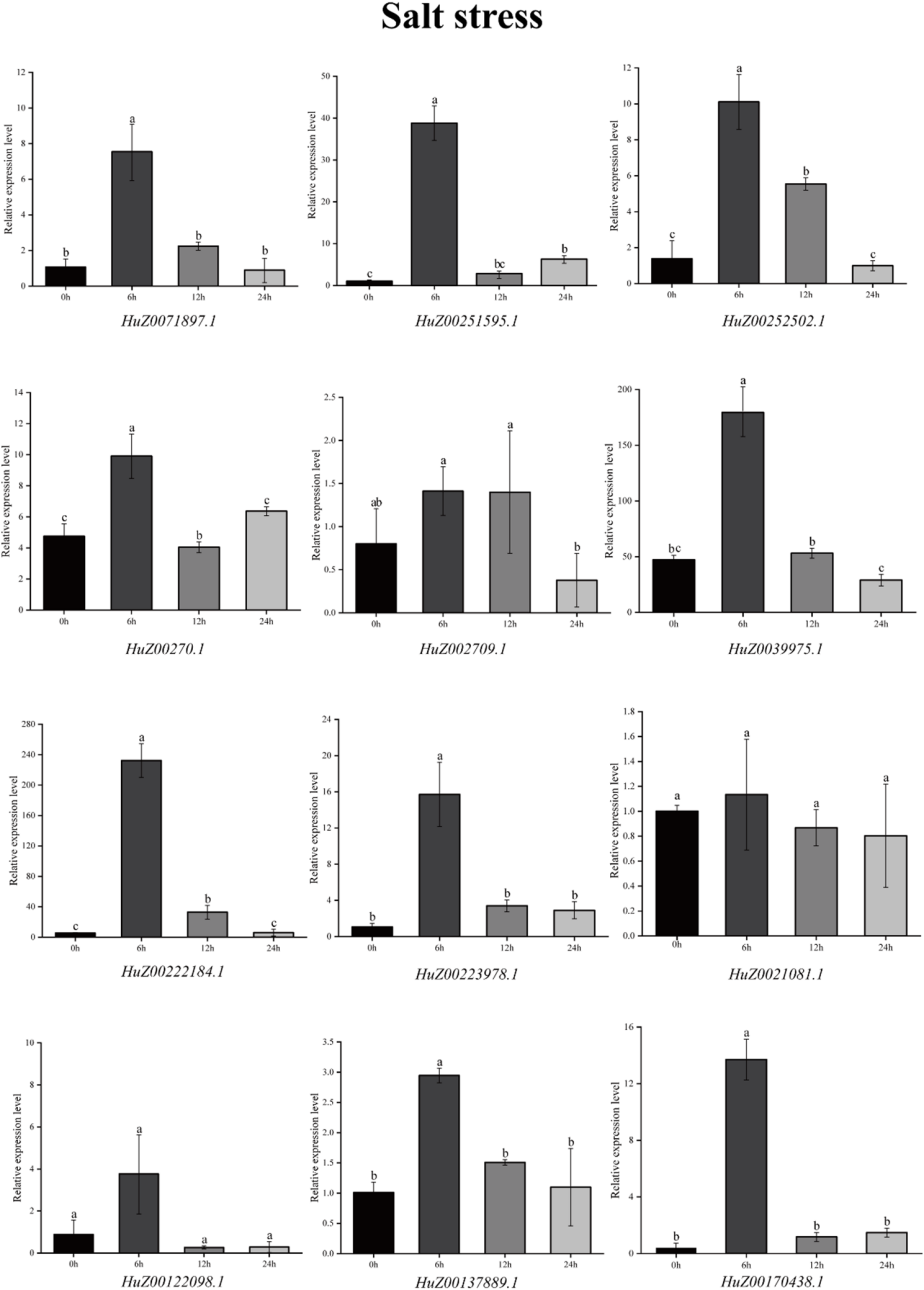
Analysis of the expression patterns of 12 candidate *RjWRKY* genes under salt stress. The error bars show the mean ± SE of three technical replicates. Letters (a, b, and c) indicate significant differences (Tukey-Kramer test, p < 0.05)

**Fig. 9:**
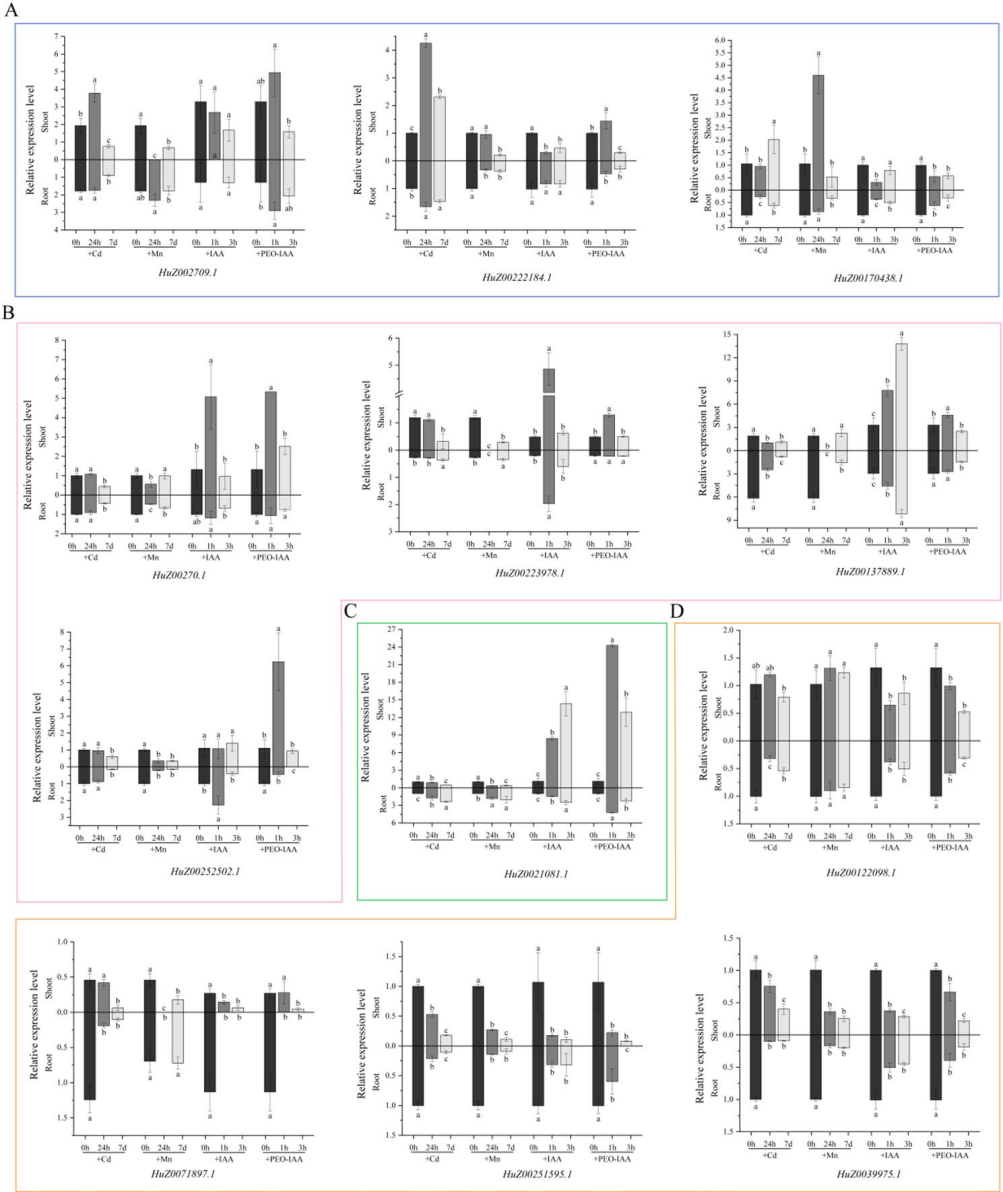
Analysis of the expression patterns of 12 *RjWRKY* genes in roots and shoots at different time points under different treatments (Cd, Mn, IAA, PEO-IAA). **A** RjWRKY genes with upregulated expression under Cd or Mn stress and downregulated expression under auxin (IAA) treatment; (**B**) RjWRKY genes that are down-regulated under Cd or Mn stress but up-regulated under auxin (IAA) treatment; (**C**) RjWRKY gene whose expression levels are upregulated under both Cd or Mn stress and auxin (IAA) treatment; (**D**) RjWRKY genes were down-regulated in the presence of both excess Cd or Mn stress and auxin (IAA). The bar chart shows the average ± SE of the three biological replicates. The letters indicate a significant difference (p < 0.05)

To investigate the role of the 12 *RjWRKY* genes in balancing the stem growth and abiotic stress response in *R. japonica*, seedlings were treated with the growth signal indole-3-acetic acid (IAA) and auxin inhibitor (PEO-IAA), NaCl, Cd, and Mn and subjected to qRT-PCR. Under salt stress, the relative expression level of all 12 *RjWRKY* genes reached the peak at 6 h in shoot. Of these, the relative expression level of nine *RjWRKY* genes at 6 h was significantly higher than that at 0, 12, and 24 h, respectively (*P* < 0.05, Fig. 8). From 6 to 24 h, the expression level of eight genes decreased continuously, but the expression level of other four genes increased following decreasing from 6 to 12 h. Conversely, high salt stress did not significantly alter the expression of the remaining three *RjWRKY* genes (*HuZ002709.1*, *HuZ0021081.1*, and *HuZ00122098.1*) (Fig. 8, Table S5). The observed expression pattern is highly suggestive of the involvement of these nine *RjWRKY* genes in the early salt stress in response of *R. japonica*. Among 12 *RjWRKY* genes, Cd or Mn exposure (24 h or 7 d), induces the expression of three *RjWRKY* genes which were downregulated by IAA compared with 0 h (CK) (Fig. 9A, Table S5). In contrast, exposure to high Cd or Mn conditions leads to the downregulation of four *RjWRKY* genes but their upregulation in IAA treatment (Fig. 9B, Table S5). Only one *RjWRKY* gene indicates increased expression level after exposure to both Cd or Mn and IAA (Fig. 9C, Table S5). The other four *RjWRKY* genes showed a decrease in expression in the presence of Cd or Mn stress and IAA or IAA-PEO (Fig. 9D, Table S5). The identified 12 *RjWRKY* genes, responsive to both auxin signals and abiotic stress, may thus represent key regulators mediating the growth-stress balance in this invasive plant species.

### *RjWRKY11* enhances Cd tolerance of transgenic *A. thaliana*

**Fig. 10:**
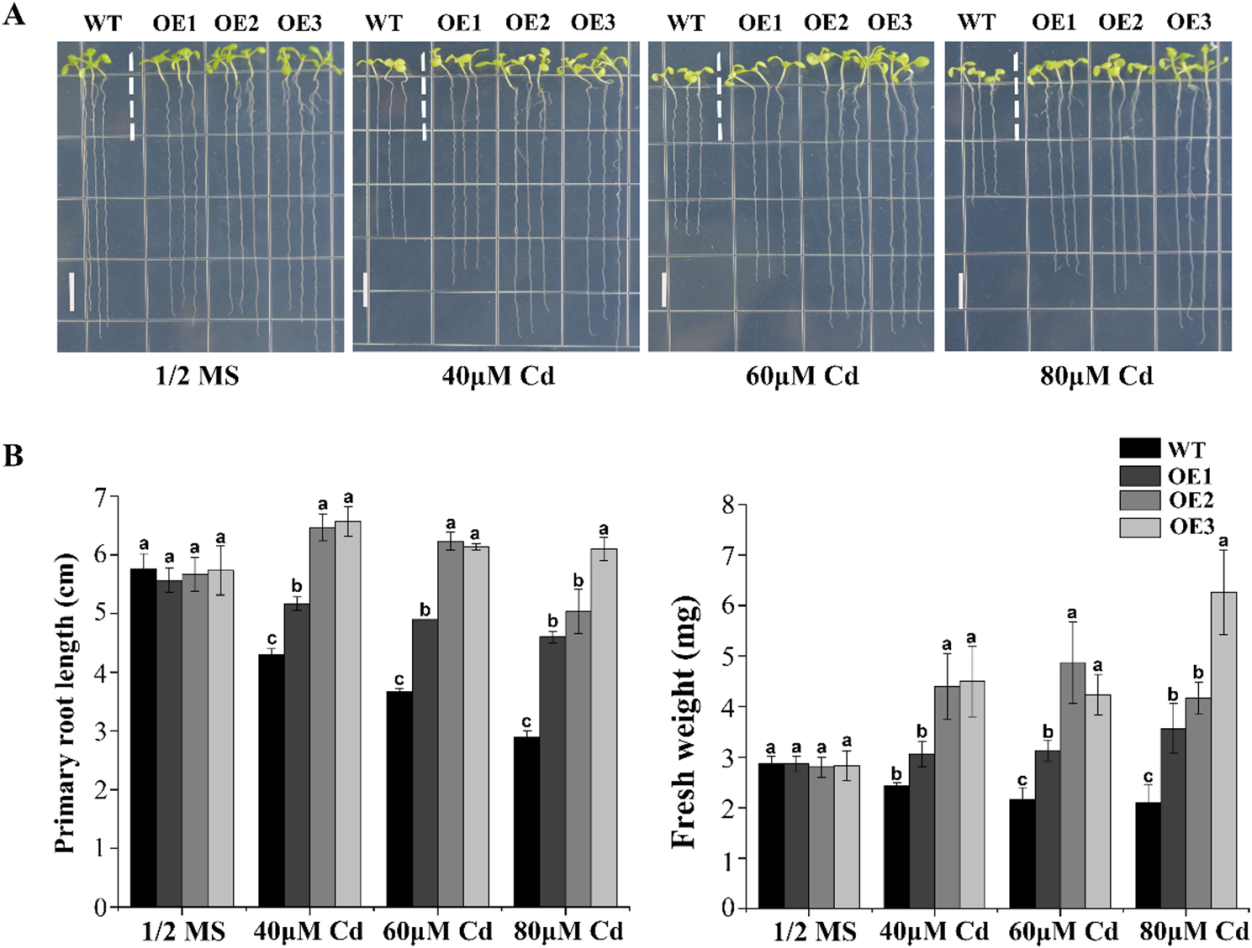
Overexpression of *RjWRKY11*enhances Cd tolerance in *A. thaliana. ***A** Transgenic and wild-type plants were grown on 1/2 MS medium with different Cd concentrations. Scale bar = 1 cm. **B** Root length and fresh weight of transgenic and wild-type plants described in (A). The error bars show the mean ± SE of three technical replicates. Letters (a, b, and c) indicate significant differences (Tukey-Kramer test, p < 0.05)

In a previous study, overexpression of *PcWRKY11* (*HuZ00251595.1*, here renamed as *RjWRKY11*) can improve the salt tolerance in *A. thaliana* [25]. To further investigate the role in Cd tolerance of *RjWRKY11* in plant, root length and fresh weight were compared of three transgenic lines (OE-1, OE-2, and OE-3) and WT on solid medium containing different concentration of Cd (control, 40 µM, 60 µM, and 80 µM). The results showed that there was no significant difference in root length and fresh weight between WT and the three transgenic lines when grown on 1/2 MS medium (Fig. 10A). By contrary, on a medium containing 40 µM Cd, WT root length was significantly inhibited compared with the transgenic lines. The inhibition was greater on the medium containing 60 µM and 80 µM Cd (Fig. 10A-B). On the exposure to Cd stress (40 µM), the fresh weight of WT was significantly reduced compared with that of the transgenic lines, and the difference was more significant with the increasing Cd concentration (60 µM and 80 µM) (Fig. 10B). These results indicate that overexpression of RjWRKY11 enhances Cd tolerance in transgenic *A. thaliana*.

## Discussion

In this study, we identified 171 RjWRKY proteins (HapA 89, HapE 82) from *R. japonica*. The number of *RjWRKY* genes on each haplotype chromosome is notably higher than that in *Ginkgo biloba* (37), *Penicillium digitatum* (47), *Betula platyphylla* (68), *A. thaliana* (74) [30–33], which may reflect large-scale gene duplication events during evolution. We detected two pairs of tandem duplications in HapA and one pair in HapE. The number of *RjWRKY* genes responding to stem elongation and UV stress in HapA is higher than that in HapE. HapE contains more *cis*-acting elements related to stress and growth and more *RjWRKY* genes responsive to MeJA treatment than HapA. It indicated that the functional divergence of RjWRKY between HapA and HapE [28].

Seven group I RjWRKYs exhibited loss of the N-terminal WRKYGQK domain, a phenomenon also documented in wheat and maize [34, 35]. We also identified specific amino acid substitutions within the WRKYGQK domain (e.g., R, Y, G, Q to K, L, E, H), resulting in variants such as WKKYGQK and WRKYEQK, similar to mutations reported in *M. dodecandrum* [36]. These alterations can impact DNA-binding specificity; for instance, the WRKYGKK variant in *NtWRKY12* enables binding to a SURE-like element instead of the canonical W-box [37], while the same variant in *GmWRKY6* and *GmWRKY21* in abolishes W-box binding [38]. These variations are predominantly found in subgroups IIc, IId, and group III, suggesting specialized functions [39]. Furthermore, The high conservation of gene structure and motif composition within RjWRKY group/subgroup indicate functional conservation, a pattern consistent with WRKY families in other plant species [40–42].

An increasing body of evidence indicates that WRKY TFs play a crucial role in trade-off between plant growth and stress resistance [43]. Our analysis revealed that a majority of *RjWRKY* promoters contain these key functional elements: 81.87% (140/171) harbor G-box elements associated with plant growth and development; 79.5% (136/171) possess ABRE elements linked to abiotic stress; and 74.26% (127/171) contain MeJA-responsive elements. Notably, the prevalence of G-boxes suggests a conserved role in growth regulation, as seen in AtWRKY2 and AtWRKY10, which participate in light signal transduction and regulation of plant growth and development via G-box binding [44]. The abundance of ABRE elements aligns with known functions of WRKYs such as GhWRKY17 and TaWRKY1, which enhance abiotic stress tolerance through ABA-mediated pathways [45, 46]. Importantly, the widespread presence of MeJA-responsive elements, combined with the observed expression changes of 154 *RjWRKY*s under MeJA treatment, strongly implies their involvement in JA signaling. JA-WRKY modules are known to coordinate growth and stress responses, a notion supported by studies showing JA pathway regulation of Cd stress response in potato [47], MeJA-induced AtWRKY45 conferring Cd tolerance [48], and WRKY factors regulating JA‑mediated development and stress responses [49]. Collectively, these findings suggest that RjWRKYs play a crucial role in coordinating JA-mediated signaling pathways during plant growth and abiotic stress response [50].

We identified 12 *RjWRKY* genes implicated in stem growth and abiotic stress. Specifically, nine were significantly upregulated at 6 h under salt stress. Among these, seven (e.g., *HuZ0039975.1*, *HuZ00251595.1*) have homologs in *A. thaliana* and other species that are established enhancers of salt tolerance [51–59]. Similarly, four *RjWRKYs* (e.g. *HuZ00223978.1*, *HuZ0071897.1*) possess homologs known to respond to drought stress (Table S6) [60, 61]. Intriguingly, these 12 *RjWRKY*s exhibited divergent expression patterns: some were induced during growth (IAA) but repressed by stress (Cd, Mn), and vice versa, while others were consistently up- or down-regulated under both conditions, suggesting their potential dual roles in coordinating growth and metal stress response. This aligns with emerging evidence that WRKY TFs often act as key nodes in cross-talk between developmental and stress-responsive pathways. For instance, AtWRKY8, AtWRKY28, and AtWRKY71 regulate both salt stress and flowering [58]; multiple members of group IId WRKYs from *A. thaliana*, balance growth and drought tolerance [62]; and group III WRKYs in *Arabidopsis* (e.g., WRKY46, WRKY54, and WRKY70) mediate this trade-off via brassinosteroid (BR)-mediated signaling [63–66]. Similarly, OsWRKY31 in rice enhances resistance by activating expression of resistance genes and JA/SA- related signaling while inhibiting auxin signaling to impede growth [67, 68].

Previous work showed that RjWRKY11 (HuZ00251595.1, formerly PcWRKY11) nucleus-localized and confers salt tolerance in *A. thaliana* [25]. Consistent with this, RjWRKY11 expression was significantly upregulated at 6 h under salt tolerance (Fig. 8). Its expression profile-upregulated during stem-elongation but downregulated by Cd, Mn and IAA, IAA-PEO (Figs. 7 and 9) suggest a role in promoting plant growth and salt tolerance, but shows an inhibitory impact of Cd and Mn stress response (Figs. 9 and 10). This resembles the function of AtWRKY7 (subgroup IId), which promotes growth while repressing drought tolerance [69]. Paradoxically, transgenic Arabidopsis overexpressing *RjWRKY11* exhibited enhanced Cd tolerance (Fig. 10). This apparent contradiction can be explained partly by a model for group IId WRKYs: their expression is repressed to further release the transcription of stress-responsive genes under stress conditions [69]. In contrast, the enhanced Cd tolerance observed in plants overexpressing *RjWRKY11* is likely mediated by alternative pathways. Under Cd stress, the overexpressed *RjWRKY11* might be modified or act independently, thus directly activating stress-responsive genes and consequently enhancing tolerance. This bypasses the typical WRKY-OBE suppression mechanism, enabling a direct launch of the tolerance program. This is supported by examples of WRKY TFs from subgroups IIa/IIc and Group III directly conferring Cd tolerance through various pathways. For instance, Arabidopsis subgroup IIa members (AtWRKY18/40/60) and AtWRKY13 (subgroup IIc) enhance Cd tolerance by binding to the W-box in the promoters of genes like *LCD*, *DCD* via the H_2_S signaling pathway [19, 70]. Within Group III WRKYs, *GmWRKY172* (soybean), ZmWRKY64 (maize), and TaWRKY70 (wheat) confers Cd tolerance in plants [71–73]. These studies highlight WRKY TFs play the pivotal role in the physiological mechanism of resistance to Cd stress [74]. We uncovered a functional paradox regarding RjWRKY11: while its expression is downregulated by Cd stress in its native *R. japonica* context consistent with its classification within group IId WRKYs that typically act as stress-response repressors within the WRKY-OBE framework [62, 69]—heterologous overexpression of RjWRKY11 in *Arabidopsis* unexpectedly enhanced Cd tolerance (Fig. 10). This contradiction challenges the direct application of the WRKY-OBE model and suggests that RjWRKY11 possesses context-dependent regulatory functions. Several testable hypotheses may explain this divergence: (1) stress-induced post-translational modifications in *Arabidopsis* could convert RjWRKY11 from a repressor to an activator [74]; (2) differential protein-protein interactions (e.g., with VQ proteins or MAP kinases) in the heterologous system may alter its transcriptional output [74, 75]; (3) overexpression may enable RjWRKY11 to activate a distinct set of Cd-responsive target genes outside its native regulatory network, analogous to known Cd-tolerance WRKYs from subgroups IIa/IIc and Group III [70–73]; and (4) dosage effects from 35 S-driven overexpression may overwhelm native repression mechanisms, unmasking latent activation capacity. These findings underscore the complexity of WRKY-mediated stress regulation and position RjWRKY11 as a context-dependent modulator whose functional versatility may contribute to the exceptional stress tolerance of *R. japonica*. Future studies employing phosphoproteomics, interactome mapping, and ChIP-seq will be essential to distinguish among these mechanistic possibilities.

## Conclusions

We identified a total of 171 *WRKY* genes in *R. japonica*, which were classified into three groups based on the number of WRKY domains and zinc finger motifs: group I (34), group II (115), and group III (22). Among these, the chromosomes HapA contains 89 genes, chromosomes HapE contains 82 genes. The comprehensive analysis of the physicochemical properties, phylogenetic relationships, chromosomal localization, conserved domains, and gene structures of RjWRKYs provides a theoretical basis for understanding the evolution of the RjWRKY gene family. Of these, 12 WRKY TFs may play a crucial balance role in stem growth in *R. japonica* and response to salt and other stress. Functional characterization of RjWRKY11, a group IId member, revealed an intriguing paradox: although its expression was downregulated by Cd stress in native *R. japonica* consistent with the WRKY-OBE repressor model [62, 69]—its heterologous overexpression in *Arabidopsis* unexpectedly enhanced Cd tolerance. This contradiction challenges the direct application of the WRKY-OBE framework and positions RjWRKY11 as a context-dependent regulator whose activity is shaped by species-specific cellular environments. We propose that this divergence may arise from mechanisms such as stress-induced post-translational modifications, differential protein interactions in heterologous systems, activation of distinct target gene sets, or dosage effects from constitutive overexpression each offering testable directions for future studies. Collectively, our findings provide a comprehensive genomic resource for *R. japonica* WRKY genes and reveal the complex regulatory nature of RjWRKY11, a feature that may contribute to the ecological success of this invasive species. The identification of growth-stress balancing candidates, particularly RjWRKY11, also offers potential genetic resources for breeding Cd-tolerant crops that maintain yield in metal-contaminated soils.

## Supplementary Information

Supplementary Material 1

## Data Availability

Data will be made available on request.
