# Supplementary material for: Genome-wide identification and functional analysis of the WRKY transcription factor family in Reynoutria japonica reveals its role in balancing growth and abiotic stress tolerance
Source: BMC Plant Biol. 2026 Mar 3;26:633. doi: 10.1186/s12870-026-08480-3 (PMC13063677; doi:10.1186/s12870-026-08480-3)
Supplement: Supplementary file 1 — Supplementary Material 1 [file 12870_2026_8480_MOESM1_ESM.docx]

**Table S1 Regulatory role of WRKY TFs in abiotic stress, plant growth and development**

| **WRKY TF** | **Plant** | **Function** | **Reference** |
| --- | --- | --- | --- |
| AtWRKY13 | *Arabidopsis thaliana* | Cd stress | [1] |
| AtWRKY25 | *Arabidopsis thaliana* | heat stress | [2] |
| AtWRKY26 | *Arabidopsis thaliana* | heat stress | [2] |
| AtWRKY33 | *Arabidopsis thaliana* | heat stress | [2] |
| AtWRKY72 | *Arabidopsis thaliana* | heat stress | [3] |
| CsWRKY26 | *Camellia sinensis* | drought stress | [4] |
| EjWRKY17 | *Eriobotrya japonica* | drought stress | [5] |
| GmWRKY13 | *Glycine max* | salt stress | [6] |
| GmWRKY17 | *Glycine max* | drought stress | [7] |
| GmWRKY21 | *Glycine max* | cold and Al stress | [6, 8] |
| GmWRKY54 | *Glycine max* | drought and salt stress | [6] |
| GmWRKY81 | *Glycine max* | Al stress | [9] |
| HvWRKY38 | *Hordeum vulgare* | drought and cold stress | [5] |
| HpWRKY85 | *Hypericum perforatum* | drought stress | [10] |
| IgWRKY50 | *Iris germanica* | drought stress | [11] |
| IgWRKY32 | *Iris germanica* | drought stress | [11] |
| LcWRKY5 | *Leymus chinensis* | drought stress | [12] |
| LlWRKY22 | *Lilium longiflorum* | heat stress | [13] |
| OsWRKY55 | *Oryza sativa* | drought stress | [14] |
| PoWRKY17 | *Paeonia ostii* | drought stress | [15] |
| PheWRKY86 | *Phyllostachys edulisis* | drought stress | [16] |
| PeWRKY1 | *Populus euphratica* | salt stress | [4] |
| PyWRKY75 | *Populus yunnanensis* | Cd stress | [17] |
| PmWRKY57 | *Prunus mume* | cold stress | [18] |
| TaWRKY31 | *Triticum aestivum* | drought stress | [19] |
| TaWRKY24 | *Triticum aestivum* | drought and salt stress | [20] |
| VvWRKY28 | *Vitis vinifera* | cold and salt stress | [21] |
| XsWRKY20 | *Xanthoceras sorbifolium* | drought stress | [22] |
| ZmWRKY58 | *Zea mays* | drought and salt stress | [23] |
| ZmWRKY64 | *Zea mays* | Cd stress | [24] |
| ZmWRKY106 | *Zea mays* | drought and heat stress | [25] |
| AtWRKY2 | *Arabidopsis thaliana* | promotes seed germination | [26] |
| AtWRKY6 | *Arabidopsis thaliana* | promotes seed germination | [27] |
| MdWRKY9 | *Malus domestica* | inhibits plant height | [28] |
| MlWRKY12 | *Miscanthus lutarioriparius* | represses stem development | [29] |
| OsWRKY29 | *Oryza sativa* | promotes seed germination | [30] |
| OsWRKY50 | *Oryza sativa* | promotes seed germination | [31] |
| PvWRKY12 | *Panicum virgatum* | represses stem development | [32] |
| TaWRKY51 | *Triticum aestivum* | promotes lateral root formation | [33] |

**Table S2 Primers for q-PCR analysis**

| **Primer Name** | **Primer Sequence (5'-3')** |
| --- | --- |
| HuZ001053.1-F | TTCATCGTCCTCATCTTCGTCG |
| HuZ001053.1-R | TCTCCACTATCCCATAATAGCCCAC |
| HuZ00252502.1-F | TCCGGAGGCGTTAGATCTGTC |
| HuZ00252502.1-R | ATCGGGATCGTCCTTGATGTC |
| HuZ00270.1-F | ACCAGTCTGCTTCATCCAGCTC |
| HuZ00270.1-R | GAGGTTGCCTTATTCTCTTTTGCAC |
| HuZ002709.1-F | GCAGCAGTGGTCTATGTGAAGCC |
| HuZ002709.1-R | CCCAATGGATTCCCTTCCACTAG |
| HuZ0039975.1-F | CTAGCCCAAATTCGGAAACCAAG |
| HuZ0039975.1-R | GGCAAAATAGACGGTACCTGGTG |
| HuZ00222184.1-F | CTTGTTCTGGAAAGCAGCGC |
| HuZ00222184.1-R | TGAACGAATTCCCAGCCGAG |
| HuZ00223978.1-F | TTCTCACCAATACCAACCACTGTAC |
| HuZ00223978.1-R | AGTACTAGAAGTCATGGCCATTTGG |
| HuZ0021081.1-F | TCAATACAGTGAAACAGGAACGTGG |
| HuZ0021081.1-R | ATATGAGTTTCCCTTGACAACCTTC |
| HuZ00122098.1-F | CCAGTTACTACCCCCTACTCCTTTG |
| HuZ00122098.1-R | TGCTGAGAGACTTCAATTGAAGGAG |
| HuZ00137889.1-F | AAGGACTCGCCTCTTTTACTGC |
| HuZ00137889.1-R | GGTCTAGTGTGATTGTGGGTGG |
| HuZ00170438.1-F | GTGCAGGTGATCATGAGAATCG |
| HuZ00170438.1-R | GAATTGGAGTCTTCAACTGGCC |
| HuZ00251595.1-F | TTGCAAAGATTTGGCCGA |
| HuZ00251595.1-R | AAATCGAGCGTCAAGGCC |

**Table S3 Analysis of physicochemical properties of RjWRKY TFs**

| Gene ID | Length (aa) | MW (Da) | pI | Instability Index | Aliphatic Index | Mean hydrophilic coefficient | Subcellular  Localization |
| --- | --- | --- | --- | --- | --- | --- | --- |
| HuZ0069995.1 | 357 | 38971.97 | 9.87 | 50.98 | 65.32 | -0.658 | Nucleus |
| HuZ0070006.1 | 357 | 38971.97 | 9.87 | 50.98 | 65.32 | -0.658 | Nucleus |
| HuZ0070333.1 | 703 | 76704.04 | 5.63 | 52.39 | 60.09 | -0.69 | Nucleus |
| HuZ0071832.1 | 425 | 47269.26 | 8.21 | 49.21 | 69.13 | -0.681 | Nucleus |
| HuZ0071865.1 | 366 | 39174.53 | 5.64 | 71.47 | 54.15 | -0.691 | Nucleus |
| HuZ0071897.1 | 317 | 34574.24 | 5.93 | 64.17 | 52.65 | -0.724 | Nucleus |
| HuZ0072609.1 | 536 | 57926.1 | 5.12 | 62.92 | 60.11 | -0.634 | Nucleus |
| HuZ0072639.1 | 519 | 55439.39 | 7.71 | 50.24 | 59.29 | -0.678 | Nucleus |
| HuZ0072945.1 | 299 | 33508.66 | 7.13 | 49.72 | 66.52 | -0.694 | Nucleus |
| HuZ00147334.1 | 315 | 34860.43 | 9.42 | 41.96 | 63.17 | -0.614 | Nucleus |
| HuZ00147472.1 | 308 | 35347.94 | 6.42 | 35.81 | 56.01 | -1.048 | Nucleus |
| HuZ00148752.1 | 537 | 59525.2 | 5.61 | 50.42 | 53.97 | -1.119 | Nucleus |
| HuZ00149045.1 | 221 | 24879.85 | 8.81 | 41.96 | 43.67 | -1.074 | Nucleus |
| HuZ00149057.1 | 314 | 35223.18 | 6.01 | 58.61 | 60.29 | -0.793 | Nucleus |
| HuZ00149157.1 | 360 | 40013.39 | 5.77 | 50.69 | 57.47 | -0.733 | Nucleus |
| HuZ00149186.1 | 131 | 14198.21 | 9.07 | 35.6 | 79.62 | -0.304 | Nucleus |
| HuZ00149187.1 | 250 | 28039.42 | 5.91 | 58.44 | 61.56 | -0.758 | Nucleus |
| HuZ00149947.1 | 592 | 64069.86 | 6.37 | 49.73 | 61.15 | -0.737 | Nucleus |
| HuZ00150190.1 | 423 | 45987.7 | 5.08 | 49.54 | 61.3 | -0.661 | Nucleus |
| HuZ00231473.1 | 405 | 45247.6 | 9.77 | 54.03 | 69.8 | -0.672 | Nucleus |
| HuZ00231722.1 | 328 | 36479.56 | 6.07 | 55.26 | 57.71 | -0.609 | Nucleus |
| HuZ00233146.1 | 666 | 73265.93 | 5.19 | 53.57 | 59.55 | -0.75 | Nucleus |
| HuZ00233443.1 | 355 | 38207.26 | 9.67 | 50.02 | 66.48 | -0.509 | Nucleus |
| HuZ00234297.1 | 365 | 39803.19 | 8.07 | 48.83 | 58.3 | -0.806 | Nucleus |
| HuZ00234544.1 | 353 | 37645 | 5.84 | 43.79 | 66.88 | -0.499 | Nucleus |
| HuZ00234651.1 | 240 | 27088.31 | 8.35 | 51.05 | 47.88 | -0.841 | Nucleus |
| HuZ00234836.1 | 353 | 38390.8 | 5.34 | 45.93 | 65.55 | -0.76 | Nucleus |
| HuZ00234900.1 | 386 | 41997.39 | 6.26 | 49.54 | 49.59 | -0.703 | Nucleus |
| HuZ0027086.1 | 336 | 36319.24 | 9.87 | 54.76 | 64.7 | -0.577 | Nucleus |
| HuZ0027158.1 | 270 | 31013.4 | 5.23 | 61.17 | 52.67 | -0.825 | Nucleus |
| HuZ0027514.1 | 172 | 19881.49 | 9.34 | 77.4 | 65.06 | -0.835 | Nucleus |
| HuZ0027800.1 | 338 | 37527.26 | 9.19 | 36.38 | 70.65 | -0.829 | Nucleus |
| HuZ0027877.1 | 261 | 28812.99 | 6.91 | 57.55 | 54.98 | -0.802 | Nucleus |
| HuZ0028125.1 | 283 | 30853.86 | 5.59 | 52.17 | 52.05 | -0.668 | Nucleus |
| HuZ0029561.1 | 199 | 22438.59 | 5.33 | 76 | 68.59 | -0.676 | Nucleus |
| HuZ0029842.1 | 370 | 40167.9 | 8.88 | 43.85 | 68.03 | -0.644 | Nucleus |
| HuZ0030101.1 | 370 | 40298.15 | 8.98 | 45.31 | 68.03 | -0.645 | Nucleus |
| HuZ00250651.1 | 306 | 33165.21 | 9.41 | 46.38 | 61.86 | -0.852 | Nucleus |
| HuZ00251184.1 | 183 | 20536.42 | 5.45 | 44.07 | 48.96 | -0.893 | Nucleus |
| HuZ00251222.1 | 312 | 34596.14 | 7.73 | 55.02 | 54.1 | -0.879 | Nucleus |
| HuZ00251566.1 | 401 | 44285.7 | 9.17 | 38.72 | 54.51 | -0.918 | Nucleus |
| HuZ00251595.1 | 337 | 36452.99 | 9.6 | 52.99 | 66.32 | -0.554 | Nucleus |
| HuZ00251715.1 | 535 | 58058.76 | 8.58 | 48.84 | 65.35 | -0.645 | Nucleus |
| HuZ00251971.1 | 283 | 30895.58 | 8.61 | 55.08 | 65.51 | -0.686 | Nucleus |
| HuZ00252266.1 | 296 | 33392.26 | 7.2 | 62.06 | 62.26 | -0.857 | Nucleus |
| HuZ00252502.1 | 355 | 38733.53 | 6.19 | 58.71 | 49.24 | -0.842 | Nucleus |
| HuZ00253072.1 | 328 | 35880.54 | 9.34 | 53.57 | 55.06 | -0.708 | Nucleus |
| HuZ00253499.1 | 442 | 48306.25 | 9.34 | 45.38 | 63.26 | -0.803 | Nucleus |
| HuZ00253699.1 | 330 | 36586.46 | 7.63 | 73.61 | 57.67 | -0.794 | Nucleus |
| HuZ00166.1 | 316 | 35046.26 | 6.33 | 41.7 | 61.77 | -0.675 | Nucleus |
| HuZ00270.1 | 298 | 32973.18 | 6.1 | 60.12 | 53.66 | -0.799 | Nucleus |
| HuZ00473.1 | 341 | 37376.88 | 6.25 | 55.48 | 62.38 | -0.648 | Nucleus |
| HuZ00533.1 | 321 | 36184.15 | 5.05 | 53.71 | 61.06 | -0.647 | Nucleus |
| HuZ002709.1 | 304 | 34507.11 | 6.4 | 39.28 | 55.49 | -0.931 | Nucleus |
| HuZ002839.1 | 324 | 35263.23 | 9.39 | 40.26 | 68.09 | -0.436 | Nucleus |
| HuZ0083140.1 | 341 | 37302.12 | 5.79 | 55.53 | 54.66 | -0.745 | Nucleus |
| HuZ0083185.1 | 350 | 38768.14 | 5.91 | 46.96 | 60 | -0.661 | Nucleus |
| HuZ0083366.1 | 571 | 63008.78 | 6.1 | 46.5 | 77.02 | -0.509 | Nucleus |
| HuZ0084108.1 | 657 | 70677.79 | 7.5 | 40.17 | 56.33 | -0.774 | Nucleus |
| HuZ0085149.1 | 354 | 39445.63 | 9.67 | 53.71 | 71.07 | -0.716 | Nucleus |
| HuZ0085256.1 | 338 | 37113.23 | 6.2 | 63.09 | 56.24 | -0.658 | Nucleus |
| HuZ0085387.1 | 349 | 39388.02 | 5.6 | 53.83 | 57.85 | -0.691 | Nucleus |
| HuZ0085420.1 | 169 | 18468.43 | 9.39 | 39.94 | 57.1 | -0.866 | Nucleus |
| HuZ0085421.1 | 315 | 34944.99 | 5.48 | 51.04 | 59.37 | -0.601 | Nucleus |
| HuZ00130781.1 | 153 | 16534.2 | 7.1 | 38.28 | 40.33 | -0.627 | Nucleus |
| HuZ00131800.1 | 399 | 45157.87 | 9.56 | 50.2 | 85.01 | -0.457 | Nucleus |
| HuZ00132363.1 | 560 | 61903.41 | 5.73 | 60.72 | 76.59 | -0.508 | Nucleus |
| HuZ00133235.1 | 351 | 39507.88 | 6.9 | 46.31 | 61.54 | -1.077 | Nucleus |
| HuZ00133312.1 | 274 | 28962.07 | 6.25 | 62.6 | 60.26 | -0.549 | Nucleus |
| HuZ00248121.1 | 175 | 20043.37 | 9.28 | 38.59 | 57.31 | -0.895 | Nucleus |
| HuZ00248374.1 | 254 | 27851.82 | 6.76 | 55.47 | 57.56 | -0.913 | Nucleus |
| HuZ00248388.1 | 460 | 49725.04 | 7.26 | 62.06 | 54.11 | -0.807 | Nucleus |
| HuZ00248831.1 | 318 | 35416.63 | 5.6 | 74.06 | 58.96 | -0.93 | Nucleus |
| HuZ00248851.1 | 308 | 34900.01 | 6.21 | 56.27 | 71.2 | -0.781 | Nucleus |
| HuZ00249474.1 | 578 | 62736.58 | 6.73 | 45.11 | 64.55 | -0.708 | Nucleus |
| HuZ00250026.1 | 301 | 34033.18 | 6.02 | 52.55 | 70.93 | -0.712 | Nucleus |
| HuZ00208806.1 | 538 | 59063.03 | 6.44 | 50.24 | 54.01 | -0.867 | Nucleus |
| HuZ00209727.1 | 348 | 38745.36 | 9.7 | 48.93 | 73.65 | -0.601 | Nucleus |
| HuZ00209827.1 | 328 | 36456.59 | 6.07 | 52.72 | 62.71 | -0.722 | Nucleus |
| HuZ00182981.1 | 294 | 32302.34 | 9.32 | 45.54 | 64.69 | -0.638 | Nucleus |
| HuZ00183581.1 | 298 | 33965.74 | 5.9 | 43.6 | 60.5 | -0.903 | Nucleus |
| HuZ00183941.1 | 481 | 53254.5 | 6.47 | 61.82 | 49.06 | -0.847 | Nucleus |
| HuZ00185305.1 | 233 | 26620.46 | 8.59 | 55.93 | 50.21 | -1.07 | Nucleus |
| HuZ00185819.1 | 752 | 81323.57 | 5.86 | 51.02 | 52.79 | -0.783 | Nucleus |
| HuZ00186177.1 | 293 | 32297.73 | 5.5 | 62.56 | 57.3 | -0.648 | Nucleus |
| HuZ00186252.1 | 232 | 26515.09 | 6.05 | 63.51 | 48.79 | -1.181 | Nucleus |
| HuZ00186279.1 | 394 | 41999.34 | 7.21 | 43.17 | 59.52 | -0.592 | Nucleus |
| HuZ00186481.1 | 573 | 62537.64 | 6.39 | 46.96 | 65.67 | -0.683 | Nucleus |
| HuZ00186625.1 | 297 | 31782.15 | 5.26 | 61.77 | 55.93 | -0.617 | Nucleus |
| HuZ0039975.1 | 359 | 38853.86 | 9.99 | 52.38 | 63.31 | -0.626 | Nucleus |
| HuZ0040296.1 | 700 | 76347.83 | 5.82 | 50.07 | 61.03 | -0.645 | Nucleus |
| HuZ0040851.1 | 360 | 39277.39 | 5.02 | 57.2 | 55.31 | -0.686 | Nucleus |
| HuZ0041866.1 | 410 | 45257.04 | 8.57 | 49.04 | 71.2 | -0.637 | Nucleus |
| HuZ0041911.1 | 484 | 52819.74 | 6.93 | 57.4 | 53.04 | -0.8 | Nucleus |
| HuZ0041932.1 | 321 | 34958.54 | 5.86 | 59.68 | 52.27 | -0.692 | Nucleus |
| HuZ0042652.1 | 519 | 55865.99 | 7.72 | 51.49 | 59.09 | -0.671 | Nucleus |
| HuZ0042851.1 | 309 | 35119.56 | 7.19 | 50.28 | 67.48 | -0.809 | Nucleus |
| HuZ00222184.1 | 366 | 40485.23 | 6.3 | 47.68 | 67.68 | -0.717 | Nucleus |
| HuZ00222322.1 | 306 | 35011.66 | 6.45 | 37.1 | 60.49 | -0.969 | Nucleus |
| HuZ00223643.1 | 516 | 56901.1 | 5.43 | 54.15 | 53.9 | -1.118 | Nucleus |
| HuZ00223970.1 | 227 | 25501.46 | 8.61 | 41.51 | 44.71 | -1.056 | Nucleus |
| HuZ00223978.1 | 314 | 35120.04 | 5.84 | 50.64 | 57.77 | -0.822 | Nucleus |
| HuZ00224082.1 | 353 | 39300.61 | 6.15 | 49.04 | 62.21 | -0.681 | Nucleus |
| HuZ00224110.1 | 92 | 9779.16 | 5.51 | 34.5 | 99.67 | -0.009 | Nucleus |
| HuZ00224111.1 | 250 | 27990.39 | 5.58 | 58.95 | 67.04 | -0.761 | Nucleus |
| HuZ00224924.1 | 592 | 64024.73 | 6.3 | 45.54 | 62.16 | -0.721 | Nucleus |
| HuZ00225155.1 | 427 | 46474.33 | 5.35 | 47.86 | 61.64 | -0.618 | Nucleus |
| HuZ0097556.1 | 436 | 48163.84 | 9.79 | 57.45 | 71.54 | -0.61 | Nucleus |
| HuZ0098955.1 | 824 | 90868.58 | 5.51 | 55.32 | 58.64 | -0.806 | Nucleus |
| HuZ0099245.1 | 348 | 37615.53 | 9.84 | 57.94 | 67.24 | -0.545 | Nucleus |
| HuZ00100164.1 | 366 | 39853.41 | 8.07 | 42.32 | 59.54 | -0.702 | Nucleus |
| HuZ00100425.1 | 341 | 36510.9 | 6.5 | 41.5 | 69.79 | -0.488 | Nucleus |
| HuZ00100430.1 | 345 | 36789.61 | 8.3 | 41.03 | 75.28 | -0.309 | Nucleus |
| HuZ00100545.1 | 313 | 35312.96 | 6.27 | 64.52 | 53.29 | -0.903 | Nucleus |
| HuZ00100762.1 | 386 | 41981.37 | 5.81 | 54.74 | 48.06 | -0.711 | Nucleus |
| HuZ0020448.1 | 338 | 36604.63 | 9.87 | 51.47 | 64.91 | -0.56 | Nucleus |
| HuZ0020545.1 | 280 | 31831.27 | 5.49 | 67.24 | 55.36 | -0.765 | Nucleus |
| HuZ0020860.1 | 172 | 20046.68 | 9.3 | 77.62 | 63.37 | -0.929 | Nucleus |
| HuZ0021081.1 | 436 | 48342.46 | 9.36 | 43.47 | 66.19 | -0.777 | Nucleus |
| HuZ0021365.1 | 223 | 24614.53 | 6.4 | 51.59 | 57.76 | -0.561 | Nucleus |
| HuZ0021687.1 | 206 | 23617.33 | 6.64 | 53.98 | 58.74 | -0.731 | Nucleus |
| HuZ0022628.1 | 340 | 38694.81 | 6.22 | 74.15 | 70.88 | -0.824 | Nucleus |
| HuZ0022881.1 | 386 | 42182.05 | 9.04 | 48.82 | 66.48 | -0.748 | Nucleus |
| HuZ00263457.1 | 307 | 34537.07 | 6.31 | 60.06 | 58.14 | -0.915 | Nucleus |
| HuZ00263504.1 | 183 | 20436.26 | 5.45 | 40.07 | 47.38 | -0.89 | Nucleus |
| HuZ00263992.1 | 305 | 33023.97 | 9.26 | 45.43 | 60.46 | -0.824 | Nucleus |
| HuZ00264326.1 | 335 | 36332.98 | 9.59 | 56.07 | 64.96 | -0.565 | Nucleus |
| HuZ00264369.1 | 469 | 51428.72 | 9.12 | 42.06 | 60.98 | -0.817 | Nucleus |
| HuZ00264821.1 | 341 | 37250.5 | 7.64 | 55.43 | 67.27 | -0.717 | Nucleus |
| HuZ00265067.1 | 303 | 34199.44 | 8.22 | 55.9 | 67.26 | -0.753 | Nucleus |
| HuZ00265288.1 | 357 | 39283.24 | 5.73 | 59.78 | 50.62 | -0.82 | Nucleus |
| HuZ00265863.1 | 306 | 33543.93 | 9.47 | 50.26 | 53.92 | -0.732 | Nucleus |
| HuZ00266252.1 | 444 | 48463.59 | 9.49 | 46.18 | 61.73 | -0.826 | Nucleus |
| HuZ00266467.1 | 327 | 36387.58 | 6.4 | 71.89 | 57.34 | -0.779 | Nucleus |
| HuZ0086277.1 | 323 | 35300.34 | 6.08 | 53.24 | 57.4 | -0.688 | Nucleus |
| HuZ0086340.1 | 292 | 32744.52 | 5.99 | 51.37 | 59.83 | -0.718 | Nucleus |
| HuZ0088548.1 | 307 | 34891.74 | 6.61 | 43.58 | 60.33 | -0.902 | Nucleus |
| HuZ0088680.1 | 326 | 35445.21 | 9.41 | 39.83 | 65.31 | -0.488 | Nucleus |
| HuZ0057791.1 | 312 | 34096.25 | 8.58 | 37.17 | 61.92 | -0.656 | Nucleus |
| HuZ0058482.1 | 344 | 37168.99 | 5.86 | 59.24 | 49.97 | -0.673 | Nucleus |
| HuZ0058533.1 | 292 | 31972.01 | 5.7 | 43.41 | 78.22 | -0.118 | Nucleus |
| HuZ0059356.1 | 522 | 56634.16 | 6.37 | 52.94 | 66.57 | -0.642 | Nucleus |
| HuZ0059369.1 | 657 | 71071.38 | 7.9 | 40.4 | 57.79 | -0.781 | Nucleus |
| HuZ0060188.1 | 383 | 42522.99 | 9.7 | 50.78 | 67.73 | -0.729 | Nucleus |
| HuZ0060260.1 | 344 | 37905.09 | 6.14 | 61.66 | 54.68 | -0.69 | Nucleus |
| HuZ0060387.1 | 340 | 37785.17 | 5.91 | 47.11 | 59.41 | -0.609 | Nucleus |
| HuZ0060419.1 | 162 | 17465.17 | 6.17 | 46.07 | 60.74 | -0.736 | Nucleus |
| HuZ0060421.1 | 320 | 35147.1 | 5.47 | 50.95 | 62.41 | -0.532 | Nucleus |
| HuZ00121397.1 | 380 | 42047.09 | 7.2 | 49.07 | 68.79 | -0.603 | Nucleus |
| HuZ00122098.1 | 471 | 52682.06 | 9.73 | 64.12 | 73.72 | -0.621 | Nucleus |
| HuZ00123441.1 | 350 | 39360.58 | 7.63 | 51.93 | 58.94 | -1.127 | Nucleus |
| HuZ00123511.1 | 286 | 30331.81 | 5.57 | 64.87 | 65.94 | -0.426 | Nucleus |
| HuZ00137132.1 | 179 | 20427.73 | 9.28 | 46.83 | 52.18 | -0.873 | Nucleus |
| HuZ00137446.1 | 325 | 34606.33 | 5.78 | 63.74 | 57.05 | -0.648 | Nucleus |
| HuZ00137889.1 | 587 | 63414.42 | 5.8 | 59.19 | 58.42 | -0.622 | Nucleus |
| HuZ00137907.1 | 308 | 34926.81 | 6.15 | 55.74 | 67.4 | -0.833 | Nucleus |
| HuZ00138566.1 | 581 | 62806.41 | 6.84 | 45.25 | 60.88 | -0.748 | Nucleus |
| HuZ00139066.1 | 301 | 33789.93 | 6.02 | 56.78 | 67.04 | -0.677 | Nucleus |
| HuZ00190412.1 | 238 | 26220.27 | 8.52 | 42.33 | 97.9 | -0.208 | Nucleus |
| HuZ00191162.1 | 542 | 59653.68 | 6.35 | 51.15 | 52.88 | -0.882 | Nucleus |
| HuZ00192037.1 | 364 | 41004.97 | 9.7 | 55.53 | 70.14 | -0.651 | Nucleus |
| HuZ00192131.1 | 326 | 36159.47 | 8.54 | 45.48 | 58.01 | -0.771 | Nucleus |
| HuZ00167659.1 | 299 | 33781.94 | 6.08 | 40.98 | 71.44 | -0.748 | Nucleus |
| HuZ00167951.1 | 484 | 53275.6 | 6.24 | 57.69 | 50.19 | -0.809 | Nucleus |
| HuZ00169524.1 | 272 | 31386.77 | 9.7 | 54.4 | 46.62 | -1.238 | Nucleus |
| HuZ00170028.1 | 737 | 79799.07 | 5.86 | 49.99 | 53.3 | -0.755 | Nucleus |
| HuZ00170379.1 | 278 | 30483.72 | 5.63 | 67 | 54.78 | -0.691 | Nucleus |
| HuZ00170438.1 | 720 | 80576.85 | 7.91 | 51.79 | 80.81 | -0.314 | Nucleus |
| HuZ00170464.1 | 434 | 46733.66 | 6.72 | 44.04 | 65.48 | -0.6 | Nucleus |
| HuZ00170670.1 | 577 | 63176.31 | 6.26 | 48.33 | 65.04 | -0.71 | Nucleus |
| HuZ00170834.1 | 297 | 31823.07 | 5.36 | 65.45 | 55.99 | -0.655 | Nucleus |

**Table S4 Distribution of *RjWRKY* members on chromosomes**

|  | Chr. 1 | | Chr. 2 | | Chr. 3 | | Chr. 4 | | Chr. 5 | | Chr. 6 | | Chr. 7 | | Chr. 8 | | Chr. 9 | | Chr. 10 | | Chr. 11 | | Total |
| --- | --- | --- | --- | --- | --- | --- | --- | --- | --- | --- | --- | --- | --- | --- | --- | --- | --- | --- | --- | --- | --- | --- | --- |
|  | A | E | A | E | A | E | A | E | A | E | A | E | A | E | A | E | A | E | A | E | A | E |  |
| Group I | 3 | 3 | 2 | 2 | 2 | 2 | 2 | 1 | 3 | 3 | 0 | 0 | 0 | 0 | 1 | 1 | 2 | 1 | 1 | 1 | 2 | 2 | 34 |
| Group IIa | 0 | 1 | 1 | 0 | 1 | 1 | 2 | 1 | 1 | 1 | 0 | 0 | 1 | 0 | 1 | 0 | 1 | 1 | 0 | 0 | 1 | 1 | 15 |
| Group IIb | 2 | 1 | 0 | 0 | 1 | 0 | 0 | 0 | 1 | 0 | 0 | 0 | 1 | 2 | 0 | 1 | 2 | 2 | 0 | 0 | 2 | 2 | 17 |
| Group IIc | 1 | 1 | 3 | 3 | 0 | 0 | 1 | 1 | 3 | 3 | 3 | 2 | 2 | 2 | 0 | 0 | 1 | 1 | 0 | 0 | 2 | 2 | 31 |
| Group IId | 2 | 1 | 1 | 1 | 2 | 2 | 2 | 2 | 2 | 2 | 1 | 1 | 1 | 1 | 2 | 1 | 0 | 0 | 1 | 2 | 1 | 0 | 28 |
| Group IIe | 0 | 1 | 3 | 3 | 1 | 1 | 1 | 1 | 1 | 1 | 1 | 2 | 2 | 1 | 1 | 0 | 0 | 0 | 0 | 0 | 2 | 2 | 24 |
| Group III | 0 | 0 | 1 | 1 | 2 | 2 | 1 | 2 | 1 | 1 | 1 | 1 | 2 | 4 | 0 | 0 | 1 | 0 | 1 | 1 | 0 | 0 | 22 |
| Total | 8 | 8 | 11 | 10 | 9 | 8 | 9 | 8 | 12 | 11 | 6 | 6 | 9 | 10 | 5 | 3 | 7 | 5 | 3 | 4 | 10 | 9 | 171 |

**Table S5 The relative expression level of 12 *RjWRKY*s under expoure of Cd, Mn and IAA, PEO-IAA**

| Gene_ID | Tissues | Treatments | Expr1 | Expr2 | Expr3 |
| --- | --- | --- | --- | --- | --- |
| *HuZ0071897.1* | Shoot | CK | 0.352574872 | 0.483462035 | 0.534709726 |
|  |  | Cd-24h | 0.390657326 | 0.389534056 | 0.472967357 |
|  |  | Cd-7d | 0.053393858 | 0.100178644 | 0.028436163 |
|  |  | Mn-24h | 1.3337E-06 | 1.16613E-06 | 8.32262E-07 |
|  |  | Mn-7d | 0.155070522 | 0.246573962 | 0.129597008 |
|  |  | CK | 0.32531549 | 0.198130925 | 0.284178965 |
|  |  | IAA-1h | 0.116767434 | 0.165853486 | 0.147905306 |
|  |  | IAA-3h | 0.085548513 | 0.079297127 | 0.026595894 |
|  |  | PEO-IAA-1h | 0.11918299 | 0.440379977 | 0.274059922 |
|  |  | PEO-IAA-3h | 0.024773002 | 0.068579082 | 0.040033105 |
|  | Root | CK | 0.835145199 | 0.73021268 | 0.52115069 |
|  |  | Cd-24h | 2.724913107 | 1.483323909 | 4.721451576 |
|  |  | Cd-7d | 0.34594027 | 3.676010194 | 0.593189197 |
|  |  | Mn-24h | 2.6411E-06 | 2.30926E-06 | 1.64811E-06 |
|  |  | Mn-7d | 0.806534355 | 0.723246049 | 0.63628034 |
|  |  | CK | 1.270422443 | 1.301828082 | 0.821549393 |
|  |  | IAA-1h | 0.00011334 | 0.0001951 | 0.000149872 |
|  |  | IAA-3h | 0.000125689 | 0.000125431 | 0.000102464 |
|  |  | PEO-IAA-1h | 0.000132408 | 0.000132514 | 0.000129286 |
|  |  | PEO-IAA-3h | 0.000154546 | 8.98456E-05 | 7.15596E-05 |
| *HuZ00252502.1* | Shoot | CK | 0.891688609 | 1.02937527 | 1.089464419 |
|  |  | Cd-24h | 0.754688633 | 1.109138962 | 1.027969956 |
|  |  | Cd-7d | 0.482927131 | 0.637566445 | 0.676801398 |
|  |  | Mn-24h | 0.43345407 | 0.419714553 | 0.191853919 |
|  |  | Mn-7d | 0.3537362 | 0.314892131 | 0.359498433 |
|  |  | CK | 0.518281585 | 1.27538073 | 1.512844779 |
|  |  | IAA-1h | 0.416860312 | 1.247475129 | 1.550600594 |
|  |  | IAA-3h | 0.863403543 | 1.611676725 | 1.742746815 |
|  |  | PEO-IAA-1h | 5.235555218 | 8.226365819 | 5.246281117 |
|  |  | PEO-IAA-3h | 0.805792198 | 0.941637455 | 1.053171749 |
|  | Root | CK | 1.065756327 | 1.066967352 | 0.879409089 |
|  |  | Cd-24h | 0.806047204 | 0.966095206 | 0.87084364 |
|  |  | Cd-7d | 0.171244045 | 0.171656584 | 0.118652904 |
|  |  | Mn-24h | 0.19098353 | 0.242624309 | 0.207289166 |
|  |  | Mn-7d | 0.173836553 | 0.14824875 | 0.129559719 |
|  |  | CK | 1.076309571 | 1.084125675 | 0.85700463 |
|  |  | IAA-1h | 2.808603376 | 2.259044336 | 1.738232149 |
|  |  | IAA-3h | 0.381259553 | 0.316857116 | 0.501463911 |
|  |  | PEO-IAA-1h | 0.330045567 | 0.569877557 | 0.446856142 |
|  |  | PEO-IAA-3h | 1.24812E-06 | 9.11032E-07 | 1.20104E-06 |
| *HuZ00270.1* | Shoot | CK | 1.134290391 | 0.925509466 | 0.952565574 |
|  |  | Cd-24h | 1.033605479 | 1.070473762 | 1.131659623 |
|  |  | Cd-7d | 0.493910023 | 0.458183689 | 0.375721478 |
|  |  | Mn-24h | 0.562983363 | 0.713577617 | 0.42295332 |
|  |  | Mn-7d | 0.978426311 | 0.848675726 | 1.147957082 |
|  |  | CK | 0.313480405 | 1.454291288 | 2.193502829 |
|  |  | IAA-1h | 1.999089739 | 6.525163255 | 6.697333722 |
|  |  | IAA-3h | 0.397029039 | 0.794728135 | 1.693932018 |
|  |  | PEO-IAA-1h | 5.329958023 | 5.337397368 | 5.329958023 |
|  |  | PEO-IAA-3h | 2.234741455 | 2.303360839 | 2.996146704 |
|  | Root | CK | 1.013825757 | 1.00098146 | 0.985395661 |
|  |  | Cd-24h | 0.777613074 | 0.949606063 | 1.004138255 |
|  |  | Cd-7d | 0.406459624 | 0.42538543 | 0.458593159 |
|  |  | Mn-24h | 0.482475947 | 0.482022635 | 0.452287799 |
|  |  | Mn-7d | 0.696873615 | 0.586825748 | 0.708563525 |
|  |  | CK | 0.876325365 | 1.067043528 | 1.069430292 |
|  |  | IAA-1h | 1.526225552 | 0.881273229 | 1.137631446 |
|  |  | IAA-3h | 0.823411348 | 0.546237533 | 0.688662451 |
|  |  | PEO-IAA-1h | 1.143921589 | 0.618842919 | 1.422044251 |
|  |  | PEO-IAA-3h | 0.781877127 | 0.713939661 | 0.82720042 |
| *HuZ002709.1* | Shoot | CK | 2.066998706 | 2.269516735 | 1.476102607 |
|  |  | Cd-24h | 3.218388359 | 4.259125078 | 3.858566056 |
|  |  | Cd-7d | 0.786595325 | 0.825368087 | 0.648054402 |
|  |  | Mn-24h | 5.958E-07 | 5.20941E-07 | 3.71794E-07 |
|  |  | Mn-7d | 0.667514348 | 0.577107395 | 0.766034386 |
|  |  | CK | 4.352355167 | 2.771999353 | 2.757896603 |
|  |  | IAA-1h | 3.830593746 | 2.728532454 | 1.490579882 |
|  |  | IAA-3h | 2.114866221 | 1.96296945 | 0.962977374 |
|  |  | PEO-IAA-1h | 5.652535659 | 5.777478607 | 3.402327669 |
|  |  | PEO-IAA-3h | 1.97214058 | 1.459558049 | 1.308690395 |
|  | Root | CK | 1.686372699 | 1.832551425 | 1.842031925 |
|  |  | Cd-24h | 1.579593014 | 1.796263099 | 1.864370557 |
|  |  | Cd-7d | 0.92744844 | 0.951621521 | 0.851256904 |
|  |  | Mn-24h | 5.958E-07 | 5.20941E-07 | 3.71794E-07 |
|  |  | Mn-7d | 0.667514348 | 0.577107395 | 0.766034386 |
|  |  | CK | 1.860139957 | 0.000759134 | 2.01976507 |
|  |  | IAA-1h | 4.10771E-05 | 4.32421E-05 | 3.97481E-05 |
|  |  | IAA-3h | 1.64987799 | 1.189089728 | 1.076624091 |
|  |  | PEO-IAA-1h | 3.029305216 | 2.376470714 | 3.307103359 |
|  |  | PEO-IAA-3h | 2.508206868 | 1.686782978 | 2.025633949 |
| *HuZ0039975.1* | Shoot | CK | 0.848983213 | 1.115262719 | 1.056145401 |
|  |  | Cd-24h | 0.771955769 | 0.844769298 | 0.649533168 |
|  |  | Cd-7d | 0.403441732 | 0.483568575 | 0.318937091 |
|  |  | Mn-24h | 0.314565497 | 0.393559467 | 0.375947724 |
|  |  | Mn-7d | 0.202655193 | 0.278477251 | 0.280889935 |
|  |  | CK | 0.971058956 | 0.99840597 | 1.03144775 |
|  |  | IAA-1h | 0.346678929 | 0.385308114 | 0.395537976 |
|  |  | IAA-3h | 0.269255084 | 0.303693239 | 0.289166765 |
|  |  | PEO-IAA-1h | 0.532143637 | 0.808057397 | 0.655393086 |
|  |  | PEO-IAA-3h | 0.213883053 | 0.191534508 | 0.253603474 |
|  | Root | CK | 0.97103114 | 1.02635667 | 1.003387146 |
|  |  | Cd-24h | 0.090693587 | 0.100307639 | 0.102252336 |
|  |  | Cd-7d | 0.093023317 | 0.091972255 | 0.08259787 |
|  |  | Mn-24h | 0.169951166 | 0.197273176 | 0.134555239 |
|  |  | Mn-7d | 0.213920664 | 0.186988523 | 0.187798161 |
|  |  | CK | 1.07260819 | 0.842839361 | 1.106150149 |
|  |  | IAA-1h | 0.582841501 | 0.46055466 | 0.471368055 |
|  |  | IAA-3h | 0.432727088 | 0.432695051 | 0.476742045 |
|  |  | PEO-IAA-1h | 0.291213929 | 0.388322108 | 0.50483469 |
|  |  | PEO-IAA-3h | 0.234739414 | 0.190868259 | 0.132076949 |
| *HuZ00222184.1* | Shoot | CK | 1.025755731 | 1.020166155 | 0.955619794 |
|  |  | Cd-24h | 4.374048425 | 4.078438915 | 4.325118208 |
|  |  | Cd-7d | 2.320995832 | 2.252124995 | 2.37070297 |
|  |  | Mn-24h | 0.909541464 | 0.857221065 | 1.103694079 |
|  |  | Mn-7d | 0.23640157 | 0.235443394 | 0.170541019 |
|  |  | CK | 0.978005997 | 1.050401334 | 0.973426617 |
|  |  | IAA-1h | 0.293954254 | 0.359378207 | 0.254362794 |
|  |  | IAA-3h | 0.404251325 | 0.344848453 | 0.632630467 |
|  |  | PEO-IAA-1h | 1.190193305 | 1.373463894 | 1.758938322 |
|  |  | PEO-IAA-3h | 0.326715285 | 0.301671197 | 0.260666883 |
|  | Root | CK | 0.921152349 | 1.103076539 | 0.984153607 |
|  |  | Cd-24h | 1.761886354 | 1.75023177 | 1.468506105 |
|  |  | Cd-7d | 1.486782674 | 1.4057958 | 1.503322436 |
|  |  | Mn-24h | 0.363939919 | 0.286161558 | 0.325545957 |
|  |  | Mn-7d | 0.338719242 | 0.347791255 | 0.427517529 |
|  |  | CK | 0.81498465 | 1.354595173 | 0.905818209 |
|  |  | IAA-1h | 0.730516521 | 0.92417101 | 0.844746961 |
|  |  | IAA-3h | 0.88896172 | 0.91543217 | 0.695661908 |
|  |  | PEO-IAA-1h | 0.485316626 | 0.550067788 | 0.360032414 |
|  |  | PEO-IAA-3h | 0.411490548 | 0.228324835 | 0.250572782 |
| *HuZ00223978.1* | Shoot | CK | 1.116722405 | 1.278208719 | 1.186972997 |
|  |  | Cd-24h | 1.075367509 | 1.123203787 | 1.151720515 |
|  |  | Cd-7d | 0.009339642 | 0.473284489 | 0.47548523 |
|  |  | Mn-24h | 1.96545E-07 | 1.7185E-07 | 1.22649E-07 |
|  |  | Mn-7d | 0.281394443 | 0.261996132 | 0.336837856 |
|  |  | CK | 0.511225834 | 0.508180647 | 0.453269513 |
|  |  | IAA-1h | 4.662715566 | 5.539439144 | 4.376871348 |
|  |  | IAA-3h | 0.681200358 | 0.655846988 | 0.550489015 |
|  |  | PEO-IAA-1h | 1.218917651 | 1.364117132 | 1.304120899 |
|  |  | PEO-IAA-3h | 0.483705621 | 0.5308596 | 0.477796 |
|  | Root | CK | 0.292986221 | 0.267976737 | 0.251965345 |
|  |  | Cd-24h | 0.30528656 | 0.302646718 | 0.262930822 |
|  |  | Cd-7d | 0.354498536 | 0.413062681 | 0.32232698 |
|  |  | Mn-24h | 7.29435E-08 | 6.37784E-08 | 4.55185E-08 |
|  |  | Mn-7d | 0.406592197 | 0.32015237 | 0.317941286 |
|  |  | CK | 0.212258524 | 0.181306935 | 0.218535854 |
|  |  | IAA-1h | 2.175200559 | 2.084158173 | 1.650511404 |
|  |  | IAA-3h | 0.891171625 | 0.43374834 | 0.472851483 |
|  |  | PEO-IAA-1h | 0.22050733 | 0.224732756 | 0.216338468 |
|  |  | PEO-IAA-3h | 0.219238287 | 0.208905855 | 0.228342948 |
| *HuZ0021081.1* | Shoot | CK | 0.982036357 | 1.081572646 | 0.941492226 |
|  |  | Cd-24h | 0.809873784 | 0.919961269 | 0.877366376 |
|  |  | Cd-7d | 0.463310577 | 0.436633732 | 0.455544585 |
|  |  | Mn-24h | 0.356215681 | 0.307352203 | 0.38257184 |
|  |  | Mn-7d | 0.49993324 | 0.320852368 | 0.33038449 |
|  |  | CK | 0.518281585 | 1.27538073 | 1.512844779 |
|  |  | IAA-1h | 8.635640844 | 8.085368156 | 8.450049443 |
|  |  | IAA-3h | 12.43912828 | 13.99598725 | 16.51146435 |
|  |  | PEO-IAA-1h | 24.48903294 | 24.00728352 | 24.30611353 |
|  |  | PEO-IAA-3h | 10.15965825 | 14.63769377 | 13.81834741 |
|  | Root | CK | 0.998803359 | 0.992517874 | 1.008745637 |
|  |  | Cd-24h | 1.598424238 | 2.150779566 | 1.495783593 |
|  |  | Cd-7d | 2.434479252 | 2.294304971 | 2.393672612 |
|  |  | Mn-24h | 1.871745924 | 1.614991322 | 2.010235151 |
|  |  | Mn-7d | 2.626914125 | 1.685928342 | 1.73601516 |
|  |  | CK | 0.923942151 | 1.03495829 | 1.045760833 |
|  |  | IAA-1h | 1.50608495 | 1.410115533 | 1.473717182 |
|  |  | IAA-3h | 2.169426013 | 2.440947479 | 2.879655187 |
|  |  | PEO-IAA-1h | 4.270970112 | 4.186951385 | 4.239068349 |
|  |  | PEO-IAA-3h | 1.771878736 | 2.552863265 | 2.409966491 |
| *HuZ00122098.1* | Shoot | CK | 1.307052935 | 0.79627801 | 0.960820147 |
|  |  | Cd-24h | 1.169116008 | 1.173017559 | 1.248790827 |
|  |  | Cd-7d | 0.720836662 | 0.882597786 | 0.76483892 |
|  |  | Mn-24h | 1.110112076 | 1.551671751 | 1.27825153 |
|  |  | Mn-7d | 1.173401707 | 1.179974387 | 1.342571201 |
|  |  | CK | 0.313480405 | 1.454291288 | 2.193502829 |
|  |  | IAA-1h | 0.699495077 | 0.544078442 | 0.69170768 |
|  |  | IAA-3h | 1.055647504 | 0.862461953 | 0.661465302 |
|  |  | PEO-IAA-1h | 1.043186891 | 0.918166612 | 1.011666519 |
|  |  | PEO-IAA-3h | 0.490366204 | 0.523511019 | 0.559925655 |
|  | Root | CK | 1.00332259 | 1.124805713 | 0.886098285 |
|  |  | Cd-24h | 0.270171734 | 0.320718917 | 0.370905342 |
|  |  | Cd-7d | 0.494121635 | 0.605006216 | 0.524284457 |
|  |  | Mn-24h | 0.760963507 | 1.063645378 | 0.876220328 |
|  |  | Mn-7d | 0.804347505 | 0.808852968 | 0.920310401 |
|  |  | CK | 0.986534067 | 1.086302163 | 0.933119508 |
|  |  | IAA-1h | 0.412790631 | 0.321075145 | 0.408195081 |
|  |  | IAA-3h | 0.622965642 | 0.508961715 | 0.390348251 |
|  |  | PEO-IAA-1h | 0.615612303 | 0.541834514 | 0.597011294 |
|  |  | PEO-IAA-3h | 0.289378127 | 0.308937763 | 0.330427007 |
| *HuZ00137889.1* | Shoot | CK | 1.581099423 | 2.222860637 | 1.862761714 |
|  |  | Cd-24h | 0.999896884 | 1.0533226 | 0.964656818 |
|  |  | Cd-7d | 0.99494268 | 1.151368835 | 1.252381937 |
|  |  | Mn-24h | 2.0502E-06 | 1.7926E-06 | 1.27937E-06 |
|  |  | Mn-7d | 2.064792754 | 1.910423605 | 2.791883386 |
|  |  | CK | 4.352355167 | 2.771999353 | 2.757896603 |
|  |  | IAA-1h | 7.059771083 | 8.426064639 | 7.83590424 |
|  |  | IAA-3h | 14.68152728 | 13.03404938 | 13.69946857 |
|  |  | PEO-IAA-1h | 4.544389462 | 4.99148185 | 4.216763973 |
|  |  | PEO-IAA-3h | 2.38960954 | 2.38996972 | 2.7344127 |
|  | Root | CK | 6.704698699 | 5.736027561 | 6.001215218 |
|  |  | Cd-24h | 2.564550813 | 2.598571234 | 2.260763293 |
|  |  | Cd-7d | 0.682016787 | 0.789244335 | 0.858487149 |
|  |  | Mn-24h | 1.40538E-06 | 1.2288E-06 | 8.7699E-07 |
|  |  | Mn-7d | 1.415381357 | 1.309563853 | 1.913789985 |
|  |  | CK | 3.550383715 | 3.156975947 | 2.165083221 |
|  |  | IAA-1h | 4.166158501 | 4.972444633 | 4.6241753 |
|  |  | IAA-3h | 8.66395935 | 7.691738865 | 8.084420414 |
|  |  | PEO-IAA-1h | 2.681764971 | 2.945606068 | 2.488424464 |
|  |  | PEO-IAA-3h | 1.410172084 | 1.410384636 | 1.61364959 |
| *HuZ00170438.1* | Shoot | CK | 0.629302914 | 1.128784216 | 1.407762297 |
|  |  | Cd-24h | 0.870129348 | 1.047250924 | 0.931006191 |
|  |  | Cd-7d | 2.062611881 | 2.579516005 | 1.451809306 |
|  |  | Mn-24h | 3.780487038 | 4.803901494 | 5.211109338 |
|  |  | Mn-7d | 0.872672317 | 0.077011993 | 0.623760654 |
|  |  | CK | 0.878679639 | 1.12009365 | 1.016050051 |
|  |  | IAA-1h | 0.196543306 | 0.29057283 | 0.445361619 |
|  |  | IAA-3h | 0.649210652 | 0.970758162 | 0.758425796 |
|  |  | PEO-IAA-1h | 0.684063049 | 0.661272939 | 0.282066996 |
|  |  | PEO-IAA-3h | 0.464873322 | 0.555189121 | 0.704899631 |
|  | Root | CK | 1.060670708 | 0.912248661 | 1.033489789 |
|  |  | Cd-24h | 0.207857687 | 0.224132104 | 0.354079321 |
|  |  | Cd-7d | 0.500683317 | 0.680159463 | 0.65810904 |
|  |  | Mn-24h | 0.930934803 | 0.911469879 | 0.745884988 |
|  |  | Mn-7d | 0.226152869 | 0.358612806 | 0.374585023 |
|  |  | CK | 0.977491951 | 0.986346704 | 1.037187352 |
|  |  | IAA-1h | 0.358793488 | 0.357442713 | 0.38709943 |
|  |  | IAA-3h | 0.475832149 | 0.575438128 | 0.463446579 |
|  |  | PEO-IAA-1h | 0.640683252 | 0.45939457 | 0.750941755 |
|  |  | PEO-IAA-3h | 0.309368284 | 0.185880598 | 0.444039373 |
| *HuZ00251595.1* | Shoot | CK | 1.00311082 | 1.032449155 | 0.965566995 |
|  |  | Cd-24h | 0.48915732 | 0.528434234 | 0.565205519 |
|  |  | Cd-7d | 0.188088711 | 0.177064395 | 0.175361192 |
|  |  | Mn-24h | 0.270278077 | 0.285910586 | 0.253368662 |
|  |  | Mn-7d | 0.128724541 | 0.137202356 | 0.071943081 |
|  |  | CK | 0.883467914 | 1.63283933 | 0.693211512 |
|  |  | IAA-1h | 0.181701112 | 0.153608867 | 0.191541767 |
|  |  | IAA-3h | 0.082135793 | 0.15661019 | 0.073214767 |
|  |  | PEO-IAA-1h | 0.171447395 | 0.2329033 | 0.266230278 |
|  |  | PEO-IAA-3h | 0.087269928 | 0.072900119 | 0.075438774 |
|  | Root | CK | 0.975367112 | 1.082041284 | 0.947519293 |
|  |  | Cd-24h | 0.269950131 | 0.180230768 | 0.190966866 |
|  |  | Cd-7d | 0.133619947 | 0.082299129 | 0.103555372 |
|  |  | Mn-24h | 0.146960158 | 0.144280155 | 0.130634643 |
|  |  | Mn-7d | 0.086564112 | 0.139229422 | 0.052540649 |
|  |  | CK | 0.930764563 | 1.160151736 | 0.926073306 |
|  |  | IAA-1h | 0.263256042 | 0.329922105 | 0.347517166 |
|  |  | IAA-3h | 0.153266514 | 0.533237111 | 0.269754624 |
|  |  | PEO-IAA-1h | 0.642108976 | 0.786796752 | 0.362435417 |
|  |  | PEO-IAA-3h | 2.21621E-07 | 1.61766E-07 | 2.13261E-07 |

**Table S6 The changes in expression levels of 12 *RjWRKY*s genes under different treatments.**

| Gene_ID | Homologous gene | Subfamily | MeJA | NaCl | Stem-elongation | Cd(R) | Mn(R) | IAA(R) | PEO-IAA(R) | Cd(S) | Mn(S) | IAA(S) | PEO-IAA(S) |
| --- | --- | --- | --- | --- | --- | --- | --- | --- | --- | --- | --- | --- | --- |
| *HuZ0021081.1* | *AtWRKY44* | Group I | 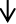 | - | 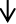 | 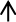 | 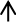 | 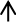 | 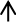 | 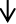 | 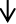 | 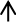 | 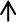 |
| *HuZ00137889.1* | *PeWRKY31* | Group IIb | 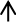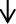 | 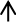 | 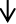 | 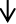 | 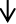 | 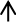 | 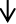 | 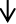 | 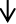 | 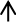 | 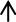 |
| *HuZ002709.1* | *TaWRKY13* | Group IIc | 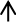 | **-** | 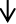 | 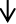 | 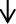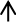 | 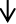 | 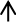 | 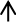 | 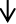 | 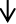 | 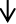 |
| *HuZ00170438.1* | *MdWRKY74* |  | 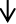 | 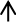 | 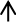 | 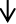 | 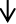 | 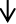 | 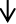 | 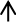 | 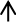 | 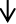 | 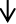 |
| *HuZ00223978.1* | *AtWRKY57* |  | 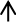 | 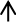 | 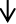 | 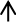 | 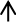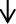 | 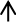 | **-** | 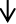 | 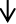 | 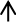 | 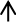 |
| *HuZ0071897.1* |  |  | 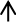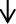 | 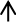 | 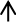 | 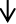 | 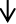 | 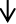 | 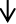 | 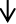 | 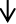 | 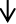 | 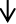 |
| *HuZ00252502.1* | *MdWRKY50* |  | 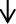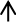 | 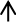 | 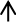 | 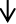 | 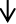 | 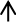 | 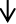 | 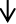 | 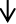 | - | 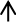 |
| *HuZ00270.1* | *AtWRKY57* |  | 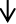 | 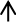 | 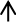 | 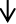 | 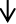 | **-** | **-** | 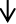 | 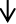 | 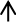 | 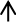 |
| *HuZ0039975.1* | *GhWRKY7* | Group IId | 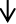 | 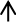 | 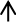 | 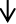 | 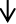 | 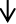 | 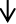 | 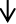 | 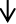 | 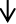 | 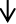 |
| *HuZ00251595.1* | *AtWRKY11* |  | 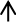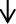 |  |  |  |  |  |  |  |  |  |  |
| *HuZ00222184.1* | *AtWRKY15* |  |  |  |  |  |  | - |  |  |  |  |  |
| *HuZ00122098.1* | *AtWRKY71* |  |  | **-** |  |  | **-** |  |  | **-** | **-** |  |  |

**Symbols indicate changes in expression level after treatment: “**-**” no significant change; significant increase; significant decrease.**

References

[1] Sheng Y, Yan X, Huang Y, et al. The WRKY transcription factor, *WRKY13*, activates *PDR8* expression to positively regulate cadmium tolerance in *Arabidopsis* [J]. Plant, Cell & Environment, 2019, 42(3): 891-903.

[2] Li S, Fu Q, Chen L, et al. *Arabidopsis thaliana WRKY25*, *WRKY26*, and *WRKY33* coordinate induction of plant thermotolerance [J]. Planta, 2011, 233: 1237-52.

[3] Cheng Z, Luan Y, Meng J, et al. WRKY transcription factor response to high-temperature stress [J]. Plants, 2021, 10(10): 2211.

[4] Chen W, Zheng C, Yao M, et al. The tea plant *CsWRKY26* promotes drought tolerance in transgenic *Arabidopsis* plants [J]. Beverage Plant Research, 2021, 1(1): 1-11.

[5] Wang D, Chen Q, Chen W, et al. A WRKY transcription factor, *EjWRKY17*, from *Eriobotrya japonica* enhances drought tolerance in transgenic *Arabidopsis* [J]. International journal of molecular sciences, 2021, 22(11): 5593.

[6] Zhou Q Y, Tian A G, Zou H F, et al. Soybean WRKY‐type transcription factor genes, *GmWRKY13*, *GmWRKY21*, *and GmWRKY54*, confer differential tolerance to abiotic stresses in transgenic *Arabidopsis* plants [J]. Plant biotechnology journal, 2008, 6(5): 486-503.

[7] Liu Y, Cao Y. Overexpression of *GmWRKY17*, a class IIb WRKY transcription factor from *Glycine max*, enhances drought tolerance in *Arabidopsis* plants [J]. Plant Growth Regulation, 2024: 1-13.

[8] Han Z, Wang J, Wang X, et al. *GmWRKY21*, a soybean WRKY transcription factor gene, enhances the tolerance to aluminum stress in *Arabidopsis thaliana* [J]. Frontiers in Plant Science, 2022, 13: 833326.

[9] Shu W, Zhou Q, Xian P, et al. *GmWRKY81* encoding a WRKY transcription factor enhances aluminum tolerance in soybean [J]. International Journal of Molecular Sciences, 2022, 23(12): 6518.

[10] Zhou W, Yang S, Yang L, et al. Genome-wide identification of the *Hypericum perforatum* WRKY Gene Family implicates *HpWRKY85* in Drought Resistance [J]. International Journal of Molecular Sciences, 2022, 24(1): 352.

[11] Zhang J, Huang D, Zhao X, et al. Drought-responsive WRKY transcription factor genes *IgWRKY50* and *IgWRKY32* from *Iris germanica* enhance drought resistance in transgenic *Arabidopsis* [J]. Frontiers in Plant Science, 2022, 13: 983600.

[12] Ma T, Li M, Zhao A, et al. *LcWRKY5*: an unknown function gene from sheepgrass improves drought tolerance in transgenic *Arabidopsis* [J]. Plant cell reports, 2014, 33: 1507-18.

[13] Wu Z, Li T, Cao X, et al. Lily WRKY factor *LlWRKY22* promotes thermotolerance through autoactivation and activation of *LlDREB2B*. Horticulture Research 9, uhac186 [Z]. 2022

[14] Huang K, Wu T, Ma Z, et al. Rice transcription factor *OsWRKY55* is involved in the drought response and regulation of plant growth [J]. International journal of molecular sciences, 2021, 22(9): 4337.

[15] Luan Y, Chen Z, Meng J, et al. *PoWRKY17* promotes drought tolerance in *Paeonia ostii* by modulating lignin accumulation [J]. Industrial Crops and Products, 2023, 204: 117228.

[16] Wu M, Zhang K, Xu Y, et al. The moso bamboo WRKY transcription factor, *PheWRKY86*, regulates drought tolerance in transgenic plants [J]. Plant physiology and biochemistry, 2022, 170: 180-91.

[17] Wu X, Chen Q, Chen L, et al. A WRKY transcription factor, *PyWRKY75*, enhanced cadmium accumulation and tolerance in poplar [J]. Ecotoxicology and Environmental Safety, 2022, 239: 113630.

[18] Wang Y, Dong B, Wang N, et al. A WRKY transcription factor *PmWRKY57* from *Prunus mume* improves cold tolerance in *Arabidopsis thaliana* [J]. Molecular Biotechnology, 2023, 65(8): 1359-68.

[19] Ge M, Tang Y, Guan Y, et al. TaWRKY31, a novel WRKY transcription factor in wheat, participates in regulation of plant drought stress tolerance [J]. BMC Plant Biology, 2024, 24(1): 27.

[20] Yu Y, He L, Wu Y. Wheat WRKY transcription factor *TaWRKY24* confers drought and salt tolerance in transgenic plants [J]. Plant Physiology and Biochemistry, 2023, 205: 108137.

[21] Liu W, Liang X, Cai W, et al. Isolation and functional analysis of *VvWRKY28*, a *Vitis vinifera* WRKY transcription factor gene, with functions in tolerance to cold and salt stress in transgenic *Arabidopsis thaliana* [J]. International Journal of Molecular Sciences, 2022, 23(21): 13418.

[22] Xiong C, Zhao S, Yu X, et al. Yellowhorn drought-induced transcription factor *XsWRKY20* acts as a positive regulator in drought stress through ROS homeostasis and ABA signaling pathway [J]. Plant Physiology and Biochemistry, 2020, 155: 187-95.

[23] Cai R, Zhao Y, Wang Y, et al. Overexpression of a maize *WRKY58* gene enhances drought and salt tolerance in transgenic rice [J]. Plant Cell, Tissue and Organ Culture (PCTOC), 2014, 119: 565-77.

[24] Gu L, Hou Y, Sun Y, et al. The maize WRKY transcription factor *ZmWRKY64* confers cadmium tolerance in *Arabidopsis* and maize (*Zea mays* L.) [J]. Plant Cell Reports, 2024, 43(2): 44.

[25] Wang C-T, Ru J-N, Liu Y-W, et al. Maize WRKY transcription factor *ZmWRKY106* confers drought and heat tolerance in transgenic plants [J]. International journal of molecular sciences, 2018, 19(10): 3046.

[26] Roscoe T J, Vaissayre V, Paszkiewicz G, et al. Regulation of *FUSCA3* expression during seed development in *Arabidopsis* [J]. Plant and Cell Physiology, 2019, 60(2): 476-87.

[27] Huang Y, Feng C-Z, Ye Q, et al. *Arabidopsis WRKY6* transcription factor acts as a positive regulator of abscisic acid signaling during seed germination and early seedling development [J]. PLoS Genetics, 2016, 12(2): e1005833.

[28] Zheng X, Zhao Y, Shan D, et al. *Md WRKY 9* overexpression confers intensive dwarfing in the M26 rootstock of apple by directly inhibiting brassinosteroid synthetase *Md DWF 4* expression [J]. New Phytologist, 2018, 217(3): 1086-98.

[29] Yu Y, Hu R, Wang H, et al. *MlWRKY12*, a novel Miscanthus transcription factor, participates in pith secondary cell wall formation and promotes flowering [J]. Plant science, 2013, 212: 1-9.

[30] Zhou C, Lin Q, Lan J, et al. WRKY transcription factor *OsWRKY29* represses seed dormancy in rice by weakening abscisic acid response [J]. Frontiers in Plant Science, 2020, 11: 691.

[31] Huang S, Hu L, Zhang S, et al. Rice *OsWRKY50* mediates ABA-dependent seed germination and seedling growth, and ABA-independent salt stress tolerance [J]. International Journal of Molecular Sciences, 2021, 22(16): 8625.

[32] Rao X, Chen X, Shen H, et al. Gene regulatory networks for lignin biosynthesis in switchgrass (Panicum virgatum) [J]. Plant Biotechnology Journal, 2019, 17(3): 580-93.

[33] Hu Z, Wang R, Zheng M, et al. *Ta WRKY 51* promotes lateral root formation through negative regulation of ethylene biosynthesis in wheat (*Triticum aestivum* L.) [J]. The Plant Journal, 2018, 96(2): 372-88.
